# Ferroptosis inhibition and mitochondrial rescue: a novel mechanism of emodin in rheumatoid arthritis

**DOI:** 10.1080/13510002.2026.2646383

**Published:** 2026-03-22

**Authors:** Linlan Zhou, Jun Liu, Jing Ren, Dehao Du, Xiaofeng Rong

**Affiliations:** a Department of Combination of Chinese and Western Medicine, The First Affiliated Hospital of Chongqing Medical University, Chongqing, People's Republic of China; b Department of Rehabilitation Medicine of Jiangbei Campus, The First Affiliated Hospital of Army Medical University, Chongqing, People's Republic of China; c College of Traditional Chinese Medicine, Chongqing Medical and Pharmaceutical College, Chongqing, People's Republic of China

**Keywords:** Rheumatoid arthritis, emodin, ferroptosis, mitochondria, oxidative stress

## Abstract

**Objectives:**

Rheumatoid arthritis (RA) is characterized by chronic synovitis and progressive joint destruction. Ferroptosis has been implicated in RA pathogenesis through synovial iron accumulation and oxidative stress. Glutathione peroxidase 4 (GPX4) and acyl-CoA synthetase long-chain family member 4 (ACSL4) are key regulators of ferroptosis, but their specific roles in RA remain incompletely defined. The objective of this research was to explore the therapeutic effects and the mechanisms behind emodin (EMO) in RA.

**Methods:**

The therapeutic efficacy and mechanisms of EMO were evaluated in collagen-induced arthritis mice and lipopolysaccharide-stimulated RAW264.7 macrophages. Joint pathology, inflammation, oxidative stress, ferroptosis, and mitochondrial function were analyzed using histology, micro-computed tomography, western blotting, immunohistochemistry, and microscopy. Key targets were identified and validated using molecular dynamics, molecular docking, proteomics, and network pharmacology.

**Results:**

EMO alleviated joint inflammation and bone destruction, reduced pro-inflammatory cytokines and oxidative stress, restored iron and mitochondrial homeostasis, and inhibited ferroptosis. Mechanistically, EMO inhibited ferroptosis through the GPX4/ACSL4 axis, as evidenced by increased GPX4 and decreased ACSL4 expression.

**Conclusions:**

EMO ameliorates experimental arthritis mainly by suppressing ferroptosis via the GPX4/ACSL4 axis, highlighting ferroptosis as a previously underappreciated therapeutic target in RA and supporting EMO as a potential adjunctive treatment for RA.

## 1. Introduction

Rheumatoid arthritis (RA) is a long-lasting autoimmune condition marked by ongoing inflammation of the synovial membrane, irregular proliferation of synovial cells, and the gradual deterioration of cartilage and bone structures, leading to intricate involvement of multiple systems [1]. As RA progresses, it can lead to joint deformities and functional impairment, significantly impacting quality of life [2]. The global prevalence of RA is approximately 0.5%–1%, with females affected approximately three times more frequently than males. The rising incidence of RA represents a growing global health concern [3].

Current RA treatments aim to control inflammation, delay disease progression, and preserve or restore joint function. However, achieving long-term stable remission remains challenging, with cartilage destruction and joint deformities common in advanced disease stages. Moreover, the extended use of medications is frequently linked to negative side effects, such as immunosuppression, gastrointestinal issues, and toxicity affecting the liver and kidneys [4]. Therefore, it is crucial to investigate more targeted and efficient treatment approaches that address new molecular mechanisms related to the pathogenesis of RA.

Recently, ferroptosis, a distinct form of programmed cell death triggered by iron metabolism imbalance and lipid peroxidation, has garnered increasing attention for its role in RA pathogenesis [5]. Within the RA synovium, a self-amplifying vicious cycle develops in which pro-inflammatory cytokines maintain an oxidative microenvironment that disrupts iron homeostasis, leading to iron overload. Subsequently, the excessive labile iron (Fe^2+^) facilitates lipid peroxidation through the Fenton reaction, directly triggering ferroptosis and further worsening mitochondrial dysfunction [6]. Mechanistically, mitochondrial dysfunction contributes to ferroptosis through several interconnected pathways: mitochondrial reactive oxygen species (mtROS) directly drive phospholipid peroxidation; dysregulated mitochondrial iron metabolism elevates cytosolic labile iron, further enhancing Fenton chemistry; and peroxidation of mitochondrial membranes (e.g. cardiolipin) amplifies lipid peroxide accumulation. Collectively, these processes suppress GPX4 activity and upregulate ACSL4 expression, establishing a self-reinforcing loop that drives ferroptosis in RA synovial cells. The resulting ferroptotic cell death and mitochondrial damage further release inflammatory mediators and oxidants, thereby re-initiating and intensifying the initial inflammatory and oxidative insults. This persistent cycle drives progressive synovial damage and joint pathology [7]. Accordingly, targeting ferroptosis regulation and mitochondrial homeostasis may represent a promising therapeutic strategy for RA.

GPX4 and ACSL4 are key regulatory factors in ferroptosis. GPX4, an antioxidant protective enzyme, can remove lipid peroxides, maintain cell membrane stability, and prevent ferroptosis [8]. In contrast, ACSL4 accelerates phospholipid peroxidation by promoting the esterification of polyunsaturated fatty acids, thereby enhancing ferroptosis [9]. In RA synovial tissues, GPX4 expression is downregulated, whereas ACSL4 is markedly upregulated, suggesting that an imbalance between these factors contributes to RA-associated ferroptosis [10,11]. Furthermore, reduced GPX4 expression or excessive ACSL4 activation can exacerbate lipid peroxidation and, through the Fenton reaction, promote reactive oxygen species (ROS) generation, thereby reinforcing a pathogenic oxidative cycle [12,13]. Regulation of the GPX4/ACSL4 axis has been shown to effectively reduce ferroptosis in neurodegenerative diseases, spinal cord injury, cerebral ischemia/reperfusion injury, and lung cancer [14–16]. However, although GPX4/ACSL4 dysregulation is associated with RA, it remains unclear how this axis precisely contributes to synovial inflammation and ferroptosis, or whether it represents a viable therapeutic target.

Emodin (EMO), a major active component of medicinal plants such as rhubarb, has attracted considerable attention due to its pleiotropic anti-inflammatory, antioxidant, and immunomodulatory effects [17]. Beyond these broad activities, accumulating evidence indicates that EMO can specifically regulate ferroptosis and mitochondrial function. In a doxorubicin (DOX)-induced cardiotoxicity model, EMO reduces ferroptosis in cardiomyocytes and alleviates DOX-induced myocardial injury [18]. EMO has been demonstrated to stimulate the signaling pathway of peroxisome proliferator-activated receptor gamma coactivator 1-alpha (PGC-1α), which helps reduce mitochondrial damage [19]. This activation promotes mitochondrial function recovery, reduces oxidative stress-induced injury, improves cell survival, and confers protective effects in chronic kidney disease. Accordingly, we hypothesize that EMO alleviates pathological damage in RA by regulating ferroptosis and improving mitochondrial function.

Current therapies for RA, such as disease-modifying antirheumatic drugs and biologics, mainly target immune activation but often fail to directly address oxidative stress and ferroptosis, which are critical mechanisms driving joint damage. Consequently, it is essential to develop therapeutic approaches that specifically focus on these pathways. To address this gap, we investigated the protective effects of EMO using both a collagen-induced arthritis (CIA) mouse model, as well as in macrophages (the central cell type in RA) to examine whether EMO mitigates pathology by specifically targeting ferroptosis and mitochondrial dysfunction. We hypothesized that pharmacological inhibition of ferroptosis by EMO attenuates synovial inflammation and joint damage, thereby clarifying the functional contribution of ferroptosis to RA pathology.

## 2. Materials and methods

### 2.1. Chemicals and reagents

Emodin (C_15_H_10_O_5_; molecular weight 270.24; purity: 99.20%) was purchased from MedChemExpress (NJ, USA, Cat. HY-14393). Methotrexate (2.5mg/tablet; Cat. 036210701) was purchased from Xinyi Ping (Shanghai, China) and provided by the First Affiliated Hospital of Chongqing Medical University (Chongqing, China). Lipopolysaccharide (LPS; Cat. L8880) was obtained from Solarbio Technology Co., Ltd. (Beijing, China). Bovine type II collagen (CII; Cat. 20021), complete Freund's adjuvant (CFA; Cat. 190288), and incomplete Freund's adjuvant (IFA; Cat. 210332) were purchased from Chondrex.

### 2.2. Establishment of CIA model in DBA/1 mice

The animal experiments complied with ethical guidelines approved by Chongqing Medical University (Ethics No. 2022-K296). Male DBA/1J mice (8–9 weeks, 20 ± 2g) were obtained from GemPharmatech Co., Ltd. (Nanjing, China). Jichui Biotech Co., Ltd. The mice were maintained in a controlled environment, which was free from specific pathogens, with conditions set at 25±2°C and a light/dark cycle of 12h each, allowing unrestricted access to food and water. On Day 0, the mice were subcutaneously injected with 100 μL of an emulsified mixture of CII and CFA for primary immunization. On day 21, a booster immunization was administered with 100 μL of the mixture of CII and IFA to establish the CIA model. The mice were randomly assigned to six groups (each group consisted of six mice) using simple randomization: control, model, EMO-L (25 mg/kg EMO), EMO-M (50 mg/kg EMO), EMO-H (100 mg/kg EMO) [20], and MTX (10 mg/kg MTX) [21]. An independent researcher prepared and concealed the randomization list until group allocation. Treatments were coded by a separate technician to ensure the blinding of personnel involved in the administration and outcome assessment. During the following 21 days, the mice were administered oral treatments based on their group allocation, with both the control and model groups receiving an equivalent volume of saline. The mice were euthanized by an overdose of sodium pentobarbital (150 mg/kg, i.p., Merck KGaA, Darmstadt, Germany; Cat. P3761). Animals meeting prespecified criteria were included, and no animals or samples were excluded.

### 2.3. Cell culture and treatment

RAW264.7 cells were purchased from the Shanghai Cell Bank, Chinese Academy of Sciences, and cultured in Dulbecco's modified Eagle medium (Thermo Fisher Scientific, Waltham, USA; Cat. C11885500BT) supplemented with 10% (v/v) fetal bovine serum (ExCell Bio, Shanghai, China; Cat. FSP500) and 100IU/mL penicillin/streptomycin (Thermo Fisher Scientific, Waltham, USA; Cat. 15140122). All the cells were cultured at 37°C in a humidified incubator containing 5% CO_2_. When the cells reached approximately 70% confluence, they were treated for 24 h as follows: control group (no treatment), LPS group (100ng/mL LPS), or LPS + EMO group (100ng/mL LPS combined with 6.25, 12.5, or 25μM EMO) [22].

### 2.4. Hematoxylin and eosin (H&E) and safranin O–fast green staining

Mouse paw joints were fixed in 4% paraformaldehyde, decalcified, and paraffin-embedded. Sections (5μm) were stained with H&E for histopathology and Safranin O–Fast Green for glycosaminoglycan (GAG) assessment in cartilage. Pathological changes were observed and recorded using an Olympus BX53 microscope.

### 2.5. Micro-computed tomography (Micro-CT) scanning and image analysis

Specimens of mouse paws were preserved in 4% paraformaldehyde for a duration of 24 h and subsequently examined with a high-resolution micro-CT system (Skyscan1276, Bruker Corporation, USA). The scanning was performed under the following conditions: a tube voltage of 55 kV, a tube current of 200 μA, an isotropic resolution of 10 μm, an aluminum filter of 0.25 mm, and a contrast setting of 0.086. Data acquisition was conducted using the Skyscan1276, followed by three-dimensional reconstruction using NRecon software and visualization through CTan software. The quantitative analysis included metrics such as the bone volume fraction/trabecular volume fraction (BV/TV), trabecular thickness (Tb.Th), and bone mineral density (BMD).

### 2.6. Enzyme-linked immunosorbent assay (ELISA) detection of inflammatory cytokines

An ELISA kit (Jiubang, China) was utilized to measure levels of inflammatory cytokines in mouse serum and cell culture supernatants, specifically interleukin-1β (IL-1β) (Cat. QZ-10247), interleukin-6 (IL-6) (Cat. QZ-10260), and tumor necrosis factor-α (TNF-α) (Cat. QZ-10225). All procedures were conducted according to the manufacturer's guidelines. Samples of serum or supernatants were introduced into microplates pre-coated with antibodies and allowed to incubate at the designated temperatures. Following this, appropriate secondary antibodies and substrate solutions were incorporated, and the enzyme reactions occurred in a dark environment. Absorbance readings were taken at 450 nm using a microplate reader (RT-6100, Rayto, China), and the concentrations of cytokines were determined using a standard curve.

### 2.7. Fe^2+^, superoxide dismutase (SOD), glutathione (GSH), and malondialdehyde (MDA) detection

The measurement of Fe^2+^, SOD, GSH, and MDA levels in RAW264.7 cells as well as mouse paw tissues was conducted utilizing the following assay kits: Fe^2+^ assay kit (Cat. Jo-1217M, Jiubang, China), GSH assay kit (Cat. BC1175, Solarbio, China), MDA assay kit (Cat. BC0025, Solarbio, China), and SOD assay kit (Cat. Jo-0101M, Jiubang, China), in accordance with the manufacturer's guidelines. After collecting and lysing cell and tissue samples, the concentrations of proteins were determined. The assays utilized colorimetric techniques, with absorbance or fluorescence readings taken using a microplate reader. Concentrations of Fe^2+^, SOD, GSH, and MDA were derived from standard curves.

### 2.8. Western blot

Proteins were extracted from tissues and cells utilizing RIPA buffer that contained 1% PMSF (Beyotime, Shanghai, China, Cat. ST506). The determination of protein concentrations was carried out using a BCA assay kit (Beyotime, Shanghai, China, Cat. P0010S). Subsequently, protein samples were loaded onto precast gels for SDS-PAGE. After the separation process, proteins were transferred to PVDF membranes and blocked with 5% skim milk (w/v) at room temperature for 1 h. After overnight incubation with primary antibodies at 4°C, membranes were washed with TBST, incubated with secondary antibodies at RT for 1 h, and visualized using an imaging system. Densitometric analysis was conducted using ImageJ software (National Institutes of Health, Bethesda, MD, USA). Antibodies and detailed protocols are provided in Supplementary Data.

### 2.9. Immunohistochemistry (IHC) analysis

Paraffin-embedded mouse hind paw sections were blocked, incubated with primary antibodies, washed, and treated with secondary antibodies for color development. Images were acquired with an Olympus BX53 microscope and analyzed using ImageJ. Antibodies and detailed protocols are in Supplementary Data.

### 2.10. Transmission electron microscopy (TEM)

RAW264.7 cells were subjected to fixation using 2.5% (v/v) glutaraldehyde and subsequently rinsed with PBS to eliminate any surplus fixative. Following this step, the samples received treatment with 1% (w/v) osmium tetroxide for a duration of 2 h, after which they underwent dehydration, embedding in resin, ultrathin sectioning, and staining procedures. The sections were then washed to remove any excess stain and dried under suitable conditions. Ultrastructural images were acquired with a transmission electron microscope (JEM-1400PLUS; JEOL, Tokyo, Japan).

### 2.11. Nitric oxide (NO) content measurement

The influence of EMO on the production of NO in LPS-stimulated RAW264.7 cells was evaluated through the Griess Reagent System. Culture supernatants were collected after EMO treatment and assayed for NO using a Griess reagent kit (Beyotime, S0021S). Briefly, 50 µL of each reagent was added to each supernatant in a 96-well plate. After a 10-min incubation, absorbance was read at 570 nm.

### 2.12. Reactive oxygen Species (ROS) level measurement

Cells were treated with varying concentrations of EMO, followed by incubation with 1µM DCFH-DA probe (S0033S, Beyotime, China) at 37°C in the dark for 30 min. Subsequently, the cells were thoroughly washed four times with PBS to eliminate any excess probes. To evaluate intracellular ROS levels, fluorescence intensity was measured using flow cytometry (CytoFLEX, Beckman Coulter, IN, USA).

### 2.13. Quantitative real-time PCR (qPCR)

Gene expression was analyzed by qPCR. Total RNA was extracted and reverse-transcribed into cDNA. Real-time PCR was performed using SYBR Green on an ABI QuantStudio system, with GAPDH as the endogenous control. Relative expression was calculated by the 2^−ΔΔCt^ method. Primer sequences and detailed protocols are listed in Supplementary Data.

### 2.14. Lipid peroxidation assay

Following treatment, RAW264.7 cells underwent incubation with 5µM BODIPY™ 581/591 C11 (D3861, Thermo Fisher) for 30 min to label lipid peroxidation. The nuclei were stained with Hoechst, and fluorescence images were quickly obtained with a DMi8 microscope (Leica, Germany).

### 2.15. Cell viability assay

RAW264.7 cells were placed in 96-well plates and incubated at 37°C in an atmosphere of 5% CO₂ for 24 h. When the cells achieved approximately 70% confluence, they were exposed to LPS (100ng/mL) along with varying concentrations of EMO (0, 6.25, 12.5, 25, 50, 100μM) for a duration of 24 h. Following this treatment, CCK-8 reagent (HY-K0301, MedChemExpress, USA) was introduced according to the manufacturer's guidance and allowed to incubate. The assessment of cell viability was conducted by measuring absorbance with a multifunctional fluorescence microplate reader (SYNERGY1, BioTek, VT, USA).

### 2.16. Mitochondrial membrane potential (MMP) assay

Following treatment with different EMO concentrations, 1 mL of JC-1 (Cat. C2006, Beyotime, China) staining solution was added to the cells and incubated at 37°C for 20 min., changes in MMP were observed using a laser scanning confocal microscope (Andor2000, UK), and the ratio of JC-1 aggregates was analyzed to assess mitochondrial function.

### 2.17. Mitochondrial reactive oxygen species (MtROS) measurement

MtROS levels in RAW264.7 cells were quantified using a MitoSox™ Red probe (Cat. M36008, Thermo Fisher, USA). Cells were treated with 1µM MitoSox Red and incubated at 37°C with 5% CO₂ for 30 min. Following incubation, fluorescence was visualized using a laser scanning confocal microscope (Andor2000, UK). MtROS levels were quantified using ImageJ software (National Institutes of Health, Bethesda, MD, USA).

### 2.18. Proteomics analysis

Protein extracts from EMO-treated RAW264.7 cells were processed by tryptic digestion and analyzed using LC - MS/MS. Differentially expressed proteins were further examined via functional enrichment analysis. Detailed protocols are listed in Supplementary Data.

### 2.19. Network pharmacology

Potential targets of EMO were predicted using databases and subsequently intersected with RA-related targets to identify overlapping candidates. The resulting set of shared targets was subjected to further analysis through Gene Ontology (GO) annotation, and Kyoto Encyclopedia of Genes and Genomes (KEGG) pathway enrichment. Detailed protocols are listed in Supplementary Data.

### 2.20. Molecular docking and molecular dynamics (MD) simulation

Based on network pharmacology and GO/KEGG pathway enrichment analyses, key targets including GPX4 and ACSL4 were selected for molecular docking and MD simulations. Detailed methods are provided in Supplementary Data.

### 2.21. Surface plasmon resonance (SPR) binding assay

SPR experiments were conducted using a Biacore T200 system (Cytiva, Sweden). Recombinant GPX4 (CSB-EP879427, Cusabio, China) and ACSL4 (URPC994Muo, Cloud-Clone Corp., China) proteins were immobilized on a CM5 sensor chip via amine coupling. EMO was injected at increasing concentrations in running buffer (1× PBS‑P⁺ with 5% (v/v) DMSO). Binding kinetics were monitored in real time and fitted to determine the equilibrium dissociation constant (*K*_D_). Detailed experimental procedures are provided in Supplementary Data.

### 2.22. Statistical analysis

All the statistical analyses and data visualization were performed using GraphPad Prism version 8.0.2 (GraphPad Software, Inc., San Diego, CA, USA). For the in vivo study, a sample size of n = 6 per group was determined based on previous similar models and pilot data. In vitro experiments were performed with at least three independent biological replicates. Prior to statistical analysis, data normality was assessed using the Shapiro–Wilk test, and homogeneity of variances was evaluated using Levene's test. For data satisfying both assumptions, one-way analysis of variance (ANOVA) followed by Tukey's post hoc test was used for comparisons among multiple groups. When data violated normality or variance assumptions, the Kruskal–Wallis test followed by Dunn's post hoc test was applied. Quantitative data are presented as mean ± standard deviation (SD) for parametric analyzes or as median with interquartile range for non-parametric analyses. A p value <0.05 was considered statistically significant.

## 3. Results

### 3.1. EMO protects joints in the CIA mouse model, maintaining joint integrity and promoting functional recovery

**Figure 1.**
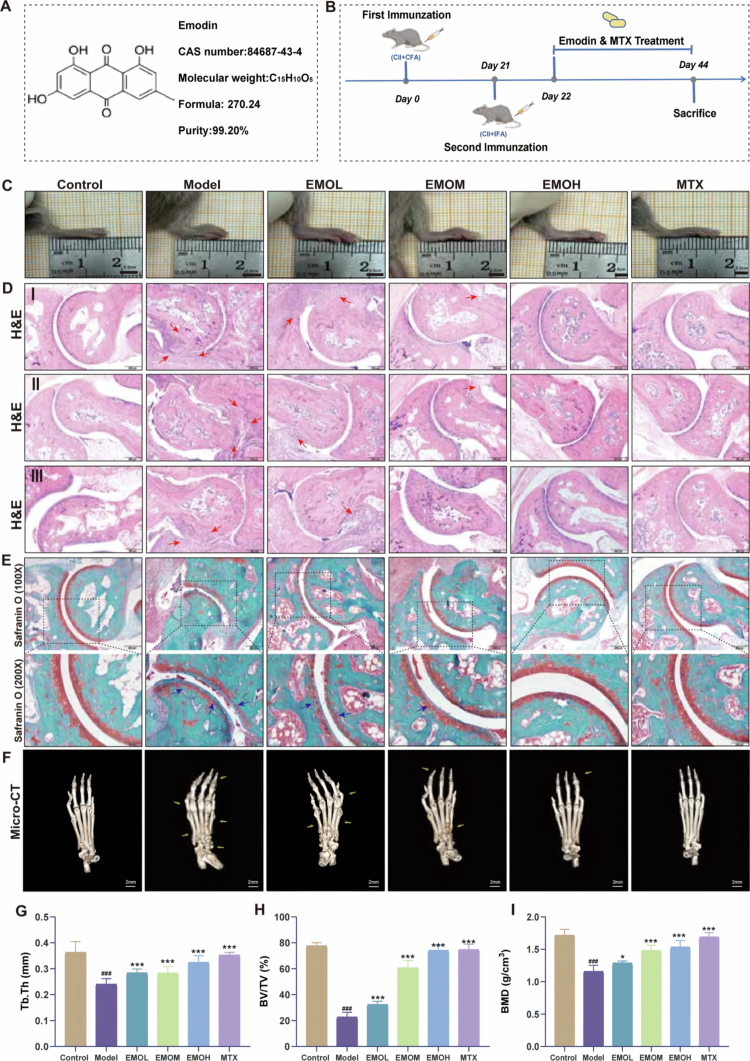
EMO improves joint histopathological changes and structural damage in CIA mice. (A) EMO's chemical structure. (B) Schematic of the animal experiment. (C) Representative photographs of the hind paws of mice in each group. (D) Representative H&E staining images of ankle joints in each group (200×); red arrow: indicates inflammatory cell infiltration. (E) Representative Safranin O–Fast Green staining images of ankle joints in each group (100×, 200×); blue arrow: indicates cartilage erosion or bone destruction. (F) Representative Micro-CT images of the hind paws of mice in each group; yellow arrows: indicates cartilage erosion or bone destruction. (G–I) Quantitative analyses of Tb.Th, BV/TV and BMD in the hind paw bones of mice (n = 6); data are presented as mean ± standard deviation (SD). ^###^*p* < 0.001 vs control group; *p < 0.05, **p < 0.01, ***p < 0.001 vs the model group. Control group: normal mouse group; Model group: CIA mouse group; EMO-L group: low-dose EMO group, 25 mg/kg; EMO-M group: medium-dose EMO group, 50 mg/kg; EMO-H group: high-dose EMO group, 100 mg/kg; MTX group: methotrexate group, 10 mg/kg; H&E: hematoxylin and eosin staining; TEM: transmission electron microscopy; Tb.Th: trabecular thickness; BV/TV: bone volume/total volume; BMD: bone mineral density.

The chemical composition of EMO is illustrated in Figure 1A, while the experimental procedure can be found in Figure 1B. Mice from the Model group demonstrated considerable redness and swelling in the ankle joints (Figure 1C). EMO treatment markedly alleviated joint swelling, particularly in the EMO-H group, displaying effects similar to those observed with MTX treatment. Histological analysis further confirmed the therapeutic effects of EMO. H&E staining (Figure 1D) and Safranin O–Fast Green staining (Figure 1E) demonstrated that the joints in the control group maintained intact structural integrity, with orderly chondrocyte arrangement and no evidence of synovial hyperplasia or inflammatory cell infiltration. Conversely, the Model group exhibited pronounced joint space narrowing, rough cartilage surfaces, extensive inflammatory cell infiltration, and synovial hyperplasia, indicating severe cartilage and bone destruction associated with RA. Following EMO treatment, these pathological alterations were significantly attenuated, with joint architecture approaching near-normal morphology.

Micro-CT was used to assess the protective effects of EMO on bone structure (Figure 1F). The Model group exhibited substantial bone erosion and destruction, characterized by rough joint surfaces, narrowed joint spaces, and multiple regions of trabecular bone erosion. In contrast, EMO and MTX treatments significantly mitigated these structural abnormalities. Quantitative analysis demonstrated that, in comparison to the Model group, the EMO group exhibited notable increases in Tb.Th, BV/TV, and BMD (Figure 1G–I), highlighting the strong bone-protective properties of EMO.

### 3.2. EMO inhibits inflammatory factor expression, improving the RA-related inflammatory microenvironment

**Figure 2.**
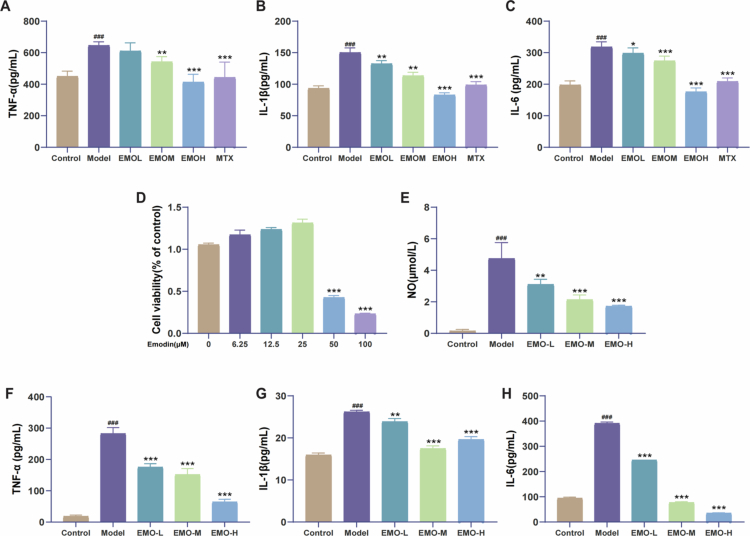
EMO alleviates inflammatory responses in CIA mice and LPS-induced RAW264.7 macrophages. (A–C) ELISA measurement of serum inflammatory factor levels (TNF-α, IL-1β, and IL-6) of mice from different groups (n = 6). (D) Cell viability assay. (E) NO levels. (F–H) ELISA measurement of inflammatory factor levels (TNF-α, IL-1β, and IL-6) in LPS-induced RAW264.7 cells (n = 3). Control group: RAW264.7 macrophage group; Model group: LPS-induced RAW264.7 macrophage group; EMO-L group: low-dose EMO group, 6.25μM EMO; EMO-M group: medium-dose EMO group, 12.5μM EMO; EMO-H group: high-dose EMO group, 25μM EMO. All data are presented as mean ± SD; ^###^*p* < 0.001 vs the control group; *p < 0.05, **p < 0.01, ***p < 0.001 vs the model group.

To assess the anti-inflammatory properties of EMO, we measured the serum concentrations of inflammatory factors in CIA mice. When compared to the Model group, the EMO group exhibited a notable decrease in serum levels of TNF-α, IL-1β, and IL-6, while the EMO-H group demonstrated a suppression effect that was similar to that observed in the MTX group (Figure 2A–C).

To confirm the anti-inflammatory effects of EMO at the cellular level, an LPS-induced RAW264.7 macrophage model was established. Cell viability assays (Figure 2D) showed that EMO concentrations of 0–25μM did not significantly affect cell viability. Accordingly, 6.25, 12.5, and 25 μM were selected as low, medium, and high doses, respectively, for subsequent experiments. LPS stimulation significantly increased NO, TNF-α, IL-1β, and IL-6 levels compared with that in the Control group, whereas EMO treatment effectively attenuated the abnormal expression of these pro-inflammatory factors (Figure 2E–H).

### 3.3. EMO alleviates oxidative stress and inhibits ferroptosis in CIA mice and LPS-induced RAW264.7 macrophages

#### 3.3.1. EMO alleviates oxidative stress and inhibits ferroptosis in CIA mice by modulating iron homeostasis

**Figure 3.**
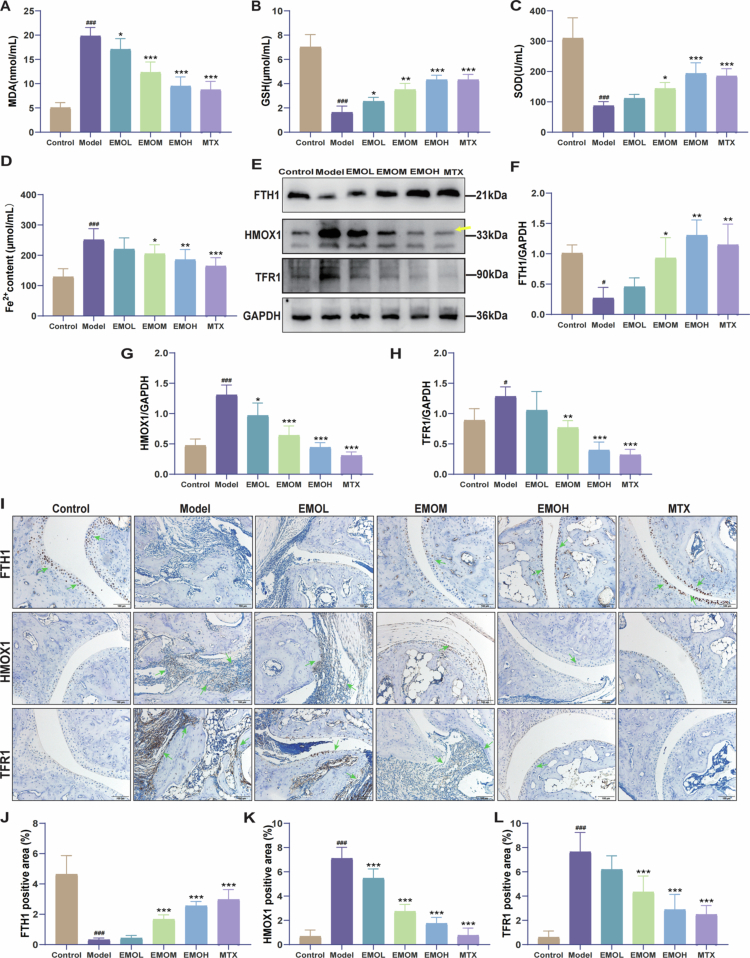
EMO improves oxidative stress and inhibits ferroptosis in the joint tissues of CIA mice. (A–C) GSH, SOD, and MDA levels (n = 6). (D) Fe^2+^ content in mouse serum (n = 6). (E–H) Representative western blot images and quantitative analysis of FTH1, HMOX1, and TFR1 in joints of CIA mice (n = 3). (I–L) Representative IHC images and quantitative analysis of FTH1, HMOX1, and TFR1 (200×, n = 6); green arrow: indicates the region of positive protein expression. All the data are presented as mean ± SD. ^#^*p *< 0.05, ^###^*p *< 0.001 vs control group; *p < 0.05, **p < 0.01, ***p < 0.001 vs the model group. Control group: normal mouse group; Model group: CIA mouse group; EMO-L group: low-dose EMO group; EMO-M group: medium-dose EMO group; EMO-H group: high-dose EMO group; MTX: methotrexate group; MDA: malondialdehyde; GSH: glutathione; SOD: superoxide dismutase; TFR1: transferrin receptor 1; HMOX1: heme oxygenase 1; FTH1: ferritin heavy chain 1.

RA pathogenesis is closely linked to oxidative stress [23]. To assess EMO's effects on oxidative stress in CIA mice, GSH, SOD, and MDA levels were measured. MDA levels were higher in the Model group than in the Control group, whereas GSH content and SOD activity showed significant decreases, suggesting considerable oxidative stress in the joint tissues affected by RA (Figure 3A–C). EMO treatment significantly reduced MDA levels and restored GSH content and SOD activity, suggesting an antioxidant effect.

To investigate the regulatory function of EMO in ferroptosis, measurements of serum-free Fe²⁺ levels were conducted. In comparison to the Model group, the EMO group exhibited a significant decrease in Fe^2+^ levels. (Figure 3D). Furthermore, the expression of ferroptosis-related proteins was analyzed by western blotting (Figure 3E–H) and IHC (Figure 3I–L). EMO treatment significantly upregulated FTH1 protein expression while downregulating HMOX1 and TFR1 compared with that those in the Model group.

#### 3.3.2. EMO protects LPS-induced RAW264.7 macrophages by alleviating oxidative stress and regulating ferroptosis

**Figure 4.**
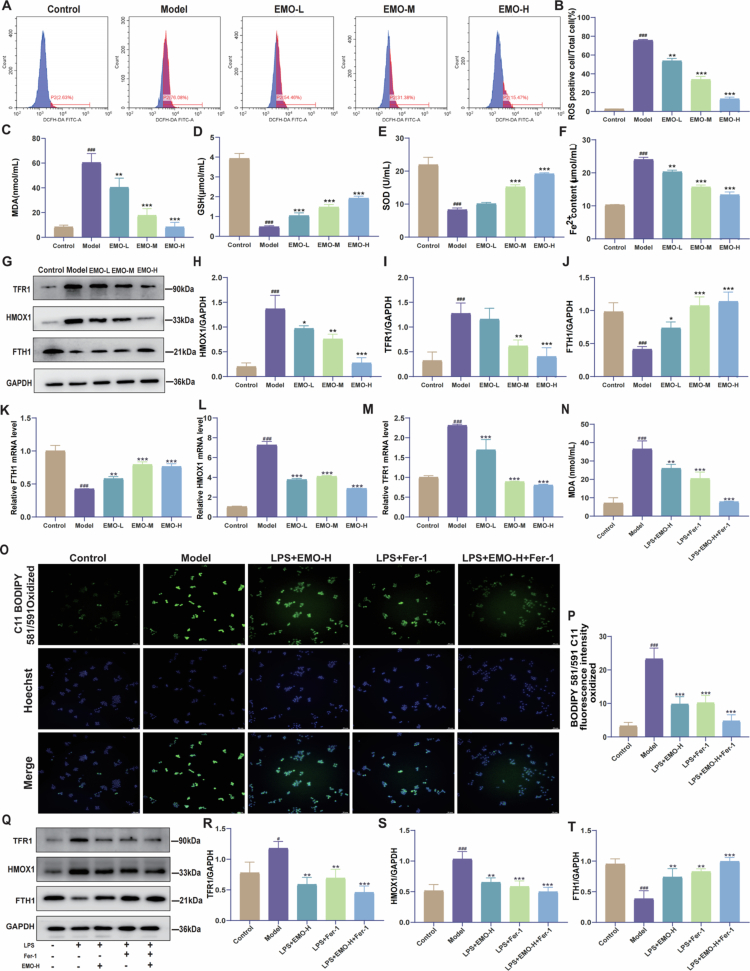
EMO alleviates macrophage oxidative stress, reduces iron ion accumulation, and regulates ferroptosis-related proteins. (A, B) Flow cytometry detection and quantitative analysis of ROS levels (n = 3). (C–E) MDA, GSH, and SOD levels in RAW264.7 cells (n = 3). (F) Measurement of Fe^2+^ levels (n = 3). (G–J) Representative western blot images and quantitative analysis of FTH1, HMOX1, and TFR1 cell expression (n = 3). (K–M) FTH1, HMOX1, and TFR1 mRNA expression (qPCR, n = 3). (N) MDA content (n = 3). (O, P) Representative fluorescent images and quantitative analysis of cells stained with C11-BODIPY (200×, n = 3). (Q–T) Western blot analysis (representative images and quantification) of TFR1, HMOX1, and FTH1 in cells treated with LPS, EMO-H, or Fer-1 (n = 3). All the data are presented as mean ± SD. ^#^*p* < 0.05,^###^*p* < 0.001 vs control group; *p < 0.05, **p < 0.01, ***p < 0.001 vs model group. Control group: RAW264.7 macrophage group; Model group: LPS-induced RAW264.7 macrophage group; EMO-L group: low-dose EMO group; EMO-M group: medium-dose EMO group; EMO-H group: high-dose EMO group; TFR1: transferrin receptor 1; HMOX1: heme oxygenase 1; FTH1: ferritin heavy chain 1.

To investigate EMO's protective effect on macrophages, an LPS-induced RAW264.7 macrophage model was used to simulate the in vitro inflammatory environment. Based on preliminary experiments, 6.25, 12.5, and 25µM EMO were applied as low, medium, and high doses, respectively. EMO treatment significantly reduced ROS content (Figure 4A and B) and MDA level (Figure 4C), while increasing GSH and SOD levels (Figure 4D–E), indicating a pronounced antioxidant effect.

Furthermore, to explore EMO's regulatory role in ferroptosis, intracellular Fe^2+^ content and ferroptosis-related protein expression were assessed. Compared with treatment in the Model group, EMO treatment significantly reduced the intracellular Fe^2+^ levels (Figure 4F). Western blot analysis (Figure 4G–J) further revealed that EMO treatment significantly upregulated FTH1 and downregulated HMOX1 and TFR1 expression. Consistently, qPCR analysis confirmed that EMO treatment increased *FTH1* mRNA levels while reducing *HMOX1* and *TFR1* mRNA levels, aligning with the observed protein-level changes (Figure 4K–M).

To further validate whether EMO's protective effects were mediated through ferroptosis inhibition, key ferroptosis-related markers were examined in the presence of the specific ferroptosis inhibitor Ferrostatin-1 (Fer-1). As shown in Figure 4O and P, Fer-1 treatment significantly attenuated LPS-induced lipid peroxidation, reflected by reduced C11-BODIPY fluorescence intensity. Consistently, MDA levels were also decreased (Figure 4N). EMO treatment suppressed lipid peroxidation to an extent comparable to that of Fer-1.

At the protein level, Western blot analysis (Figure 4Q–T) revealed that Fer-1 downregulated TFR1 and HMOX1 expression while upregulating FTH1 expression compared with that in the Model group. Similarly, EMO treatment decreased TFR1 and HMOX1 expression and increased FTH1 expression in a dose-dependent manner.

Together, these results demonstrate that EMO effectively inhibits lipid peroxidation and modulates ferroptosis-related proteins in a pattern similar to a canonical ferroptosis inhibitor, supporting the conclusion that EMO exerts its protective effects, at least in part, through suppression of ferroptosis.

### 3.4. EMO improves LPS-induced mitochondrial dysfunction in RAW264.7 macrophages

**Figure 5.**
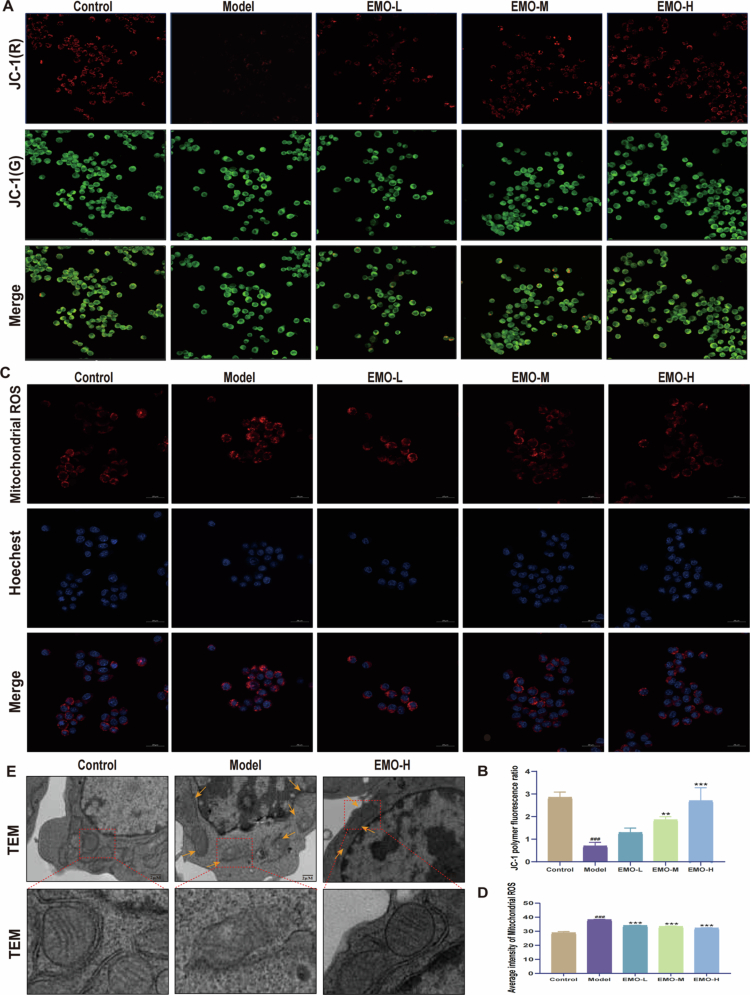
EMO improves mitochondrial dysfunction in LPS-induced RAW264.7 macrophages. (A, B) Representative fluorescence images and quantitative analysis of MMP detected by JC-1 staining (200×, n = 3). (C, D) Representative fluorescence images and quantitative analysis of mtROS levels detected by MitoSOX Red staining (200×, n = 3). (E) Representative TEM images of cells; orange arrows: indicate key ultrastructural changes (mitochondrial rupture of the outer membrane and loss of cristae structure) (scale bar = 2 μm). Data are expressed as mean ± SD. ^###^*p* < 0.001 compared with the control group; **p < 0.01, ***p < 0.001 compared with the model group. Control group: RAW264.7 macrophages; Model group: LPS-induced RAW264.7 macrophages; EMO-L group: low-dose EMO group; EMO-M group: medium-dose EMO group; EMO-H group: high-dose EMO group; TEM: transmission electron microscopy; MMP: mitochondrial membrane potential; mtROS: mitochondrial reactive oxygen species.

Mitochondria play a critical role in maintaining cellular homeostasis and regulating programmed cell death [24]. To assess EMO's effect on LPS-induced mitochondrial dysfunction in RAW264.7 macrophages, MMP and mtROS levels were measured. JC-1 fluorescence staining (Figure 5A and B) revealed that EMO treatment significantly enhanced MMP, preserving mitochondrial transmembrane potential stability. MitoSOX fluorescence probe detection (Figure 5C and D) indicated that EMO treatment markedly reduced mtROS levels, alleviating LPS-induced mitochondrial oxidative damage. TEM revealed substantial mitochondrial injury in LPS-induced cells, including rupture of the outer membraneand loss of cristae structure (Figure 5E). EMO treatment significantly improved mitochondrial morphology, restoring outer membrane integrity, and preserving cristae structure. These findings suggest that EMO stabilizes mitochondrial structure and function, mitigating LPS-induced mitochondrial damage.

### 3.5. EMO modulates ferroptosis, inflammation, and immune pathways in RA and LPS-induced macrophage damage: insights from network pharmacology and proteomics

#### 3.5.1. Network pharmacology analysis

**Figure 6.**
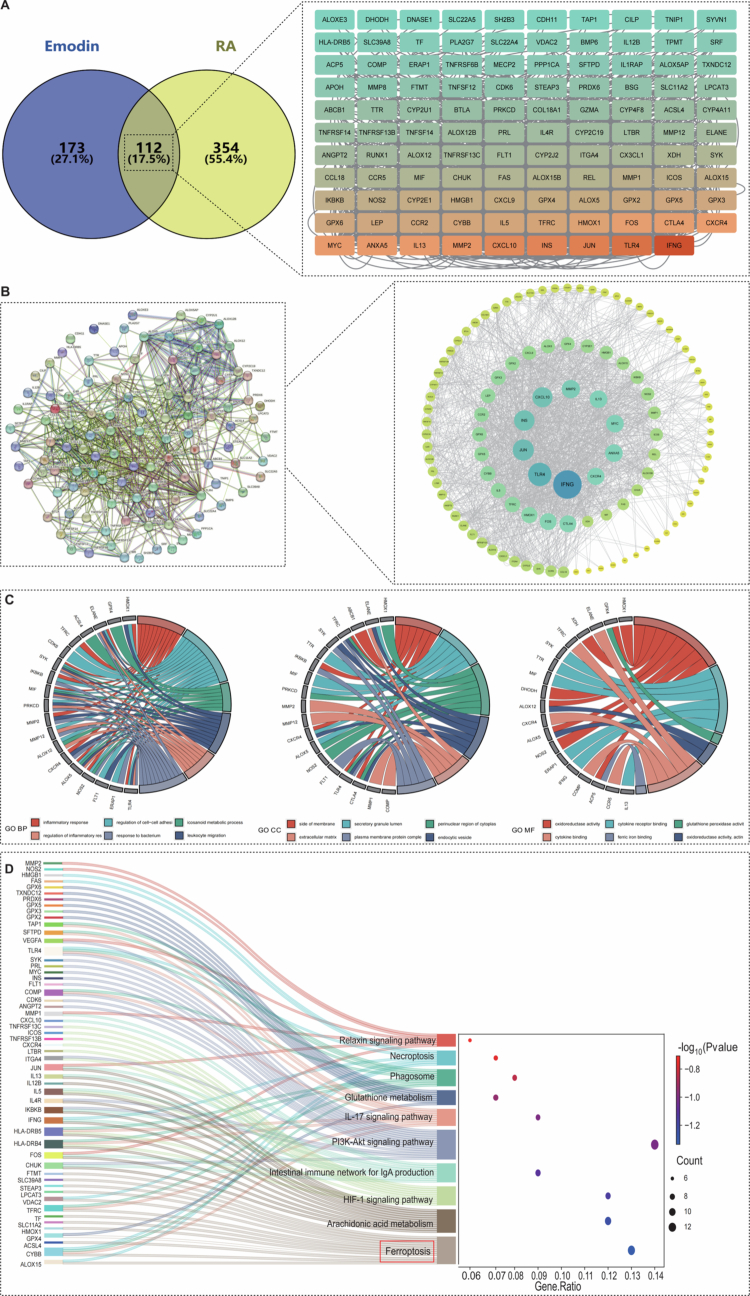
Network pharmacology analysis. (A) Venn diagram of the shared targets between EMO and RA. (B) PPI network of the shared targets between EMO and RA visualized using Cytoscape. Circular nodes represent core target proteins, with node size reflecting their topological importance in the network. (C) GO enrichment analysis, including biological processes, cellular components, and molecular functions, showing the functional enrichment of shared targets in different biological processes. (D) KEGG enrichment analysis, revealing key signaling pathways related to RA and ferroptosis. EMO: emodin; RA: rheumatoid arthritis; PPI: protein–protein interaction; GO: Gene Ontology; KEGG: Kyoto Encyclopedia of Genes and Genomes.

To provide deeper insights into the mechanism of EMO in the progression of RA, a network pharmacology strategy was utilized to systematically analyze the target genes associated with both EMO and RA. A total of 285 EMO-related targets and 466 RA-related targets were identified. Intersection analysis revealed 112 potential EMO targets. Key target screening highlighted several core proteins associated with ferroptosis (Figure 6A). Subsequently, we constructed a PPI network and performed a visual analysis using Cytoscape (Figure 6B). Node size reflected network centrality, and top-ranked core targets included ferroptosis-related proteins as well as inflammation- and immune-regulatory proteins. GO and KEGG enrichment analyzes of EMO and RA common targets were conducted to explore their potential biological functions and associated signaling pathways. GO enrichment analysis (Figure 6C) showed that EMO primarily targets the plasma membrane, cytoplasm, and extracellular space, and modulates biological processes such as inflammation, immune regulation, and signal transduction. KEGG enrichment analysis (Figure 6D) revealed EMO's involvement in key RA-related signaling pathways, including metabolic pathways, cytokine–cytokine receptor interactions, and ferroptosis-related pathways. Notably, the ferroptosis signaling pathway is recognized as critical in RA progression.

#### 3.5.2. Proteomics analysis reveals the potential mechanism of EMO in improving LPS-induced RAW264.7 macrophage damage

**Figure 7.**
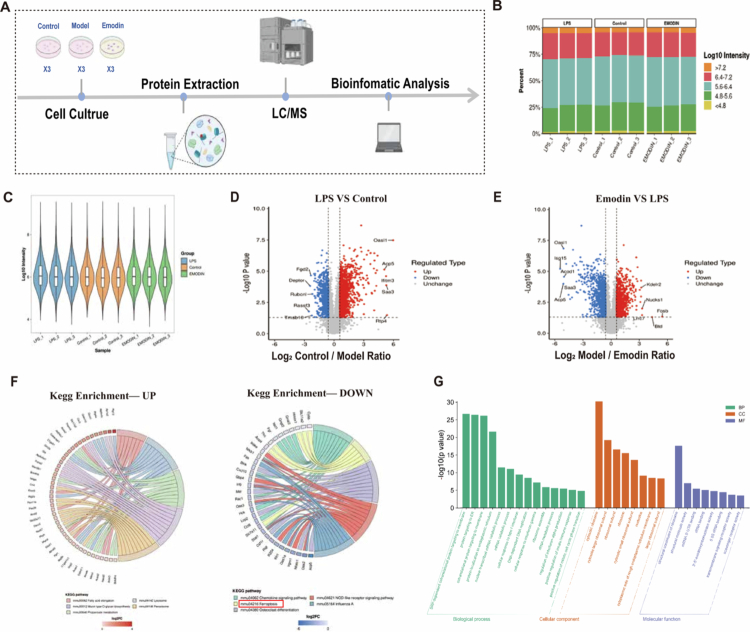
Proteomics analysis reveals the potential mechanism of EMO in improving LPS-induced RAW264.7 macrophage damage. (A) Schematic diagram of the proteomics analysis workflow. (B) Stacked bar chart of Log10 intensity distribution in each group. (C) Violin plot of the balanced distribution of protein expression intensity. (D, E) Volcano plots of DEPs between different treatment groups. (F) KEGG enrichment analysis reveals the main biological pathways regulated by EMO, particularly the ferroptosis signaling pathway. (G) GO enrichment analysis results.

To explore the mechanism of EMO in LPS-induced RAW264.7 macrophage damage, proteomic analysis was performed to investigate protein expression profiles in EMO-treated cells. The proteomics workflow is presented in Figure 7A, followed by data quality control analysis. The stacked bar chart (Figure 7B) illustrates the distribution of log10 intensity values across each group, while the violin plot (Figure 7C) demonstrates a balanced distribution of protein expression intensities, indicating good data reproducibility and reliability. A total of 7660 proteins were identified. Among these, 2008 and 1733 DEPs were identified in the comparisons between the control and model (LPS) groups and between the model and high-dose EMO groups, respectively. In comparison to the Model group, the EMO group exhibited 971 downregulated and 762 upregulated DEPs. Volcano plots (Figure 7D and E) visually demonstrated the significant regulatory effects of EMO on the global protein expression profile. To further elucidate the biological functions influenced by EMO, GO and KEGG enrichment analyses were performed on the identified DEPs. GO enrichment analysis (Figure 7G) indicated that EMO may protect cells from LPS-induced damage by regulating ribosome function, RNA metabolism, protein synthesis, and immune/inflammatory responses. KEGG pathway analysis (Figure 7F) showed that EMO was primarily enriched in the NOD-like receptor, chemokine, and ferroptosis signaling pathways, suggesting that EMO exerts anti-inflammatory effects by modulating immune- and ferroptosis-related pathways.

### 3.6. EMO may exert therapeutic effects on RA through regulation of the GPX4/ACSL4-mediated ferroptosis pathway

#### 3.6.1. Molecular docking and MD simulations reveal the therapeutic effects of EMO on RA

**Figure 8.**
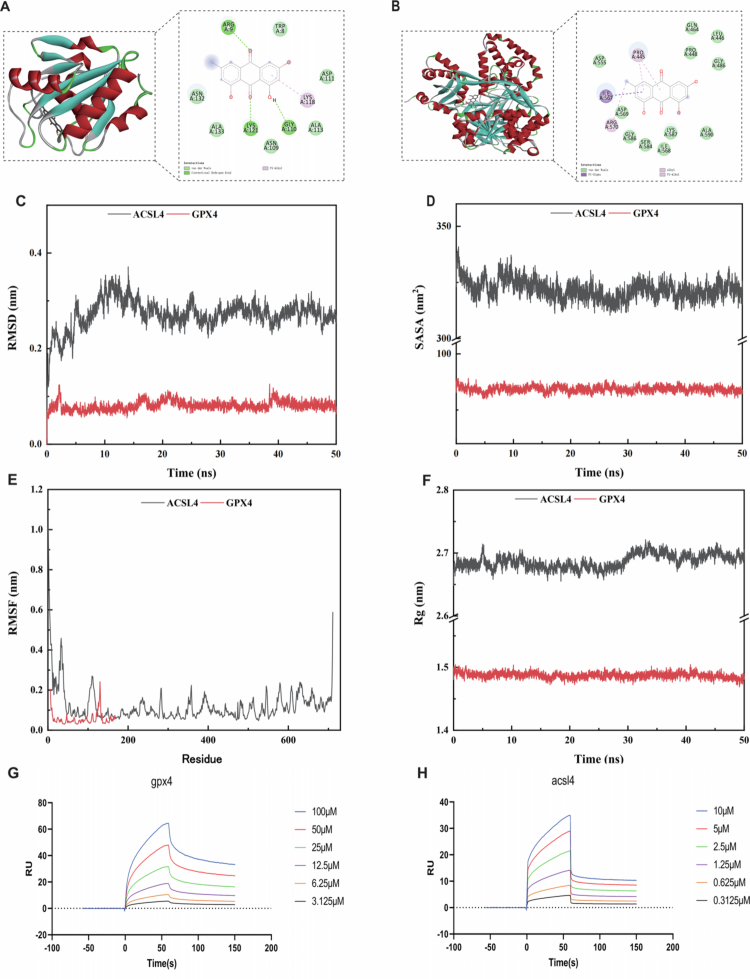
Molecular docking and molecular dynamics simulation analyzes. (A–B) Schematic of EMO's molecular docking with GPX4 and ACSL4. (C) RMSD curve of the protein–ligand complexes. (D) SASA curve of the protein–ligand complexes. (E) RMSF curve of the protein–ligand complexes. (F) Rg curve of the protein–ligand complexes. (G, H) SPR detection of the direct interaction between EMO and recombinant GPX4 and ACSL4. MD, molecular dynamics simulation; EMO, emodin; RA, rheumatoid arthritis; GPX4, glutathione peroxidase 4; ACSL4, acyl-CoA synthetase long-chain family member 4; RMSD, root mean square deviation; SASA, solvent-accessible surface areas; RMSF, root mean square fluctuation; Rg, radius of gyration.

Based on the network pharmacology and proteomics results, the GPX4/ACSL4-mediated ferroptosis pathway was selected for further validation using molecular docking, MD simulations, and SPR. Molecular docking analysis predicted the binding affinity between EMO and the target proteins GPX4 and ACSL4. The calculated binding energies indicated that EMO binds GPX4 and ACSL4 with values of −6.5 and −7.8 kcal/mol, respectively (Figure 8A and B), suggesting stable molecular interactions with both targets.

To enhance the assessment of the binding stability for the EMO–GPX4 and EMO–ACSL4 complexes, molecular dynamics (MD) simulations were performed, and the stability of the system was evaluated through root mean square deviation (RMSD) analysis(Figure 8C). The simulations were performed for 50 ns, and the RMSD trajectories indicated that both complexes reached stable equilibrium after approximately 30 ns and remained stable for the remainder of the simulation, indicating sustained binding stability. Solvent-accessible surface area (SASA) analysis showed a gradual decrease in SASA values over time (Figure 8D), reflecting increased structural compactness upon EMO binding. RMSF analysis revealed low conformational flexibility in both complexes, with average fluctuation values of 0.1202 nm for EMO–ACSL4 and 0.0560 nm for EMO–GPX4 (Figure 8E), indicating that key residues did not undergo substantial conformational changes following EMO binding. Consistently, the radius of gyration (Rg) decreased progressively in both complexes (Figure 8F), further supporting enhanced structural compactness and stable complex formation.

To experimentally validate the interactions between EMO and the ferroptosis-related proteins GPX4 and ACSL4, we performed SPR analysis (Figure 8G and H). The results demonstrated direct and dose-dependent binding between EMO and both proteins. EMO bound to GPX4 with a dissociation constant (*K*_D_) of 5.28 × 10⁻⁵ M, whereas its interaction with ACSL4 showed higher affinity, with a *K*_D_ of 2.67 × 10⁻⁶ M. Collectively, these SPR findings confirm stable protein-level interactions between EMO and GPX4/ACSL4, supporting the involvement of EMO in regulating the ferroptosis pathway.

#### 3.6.2. EMO inhibits ferroptosis in RA by regulating the GPX4/ACSL4 axis: in Vivo and in vitro validation

**Figure 9.**
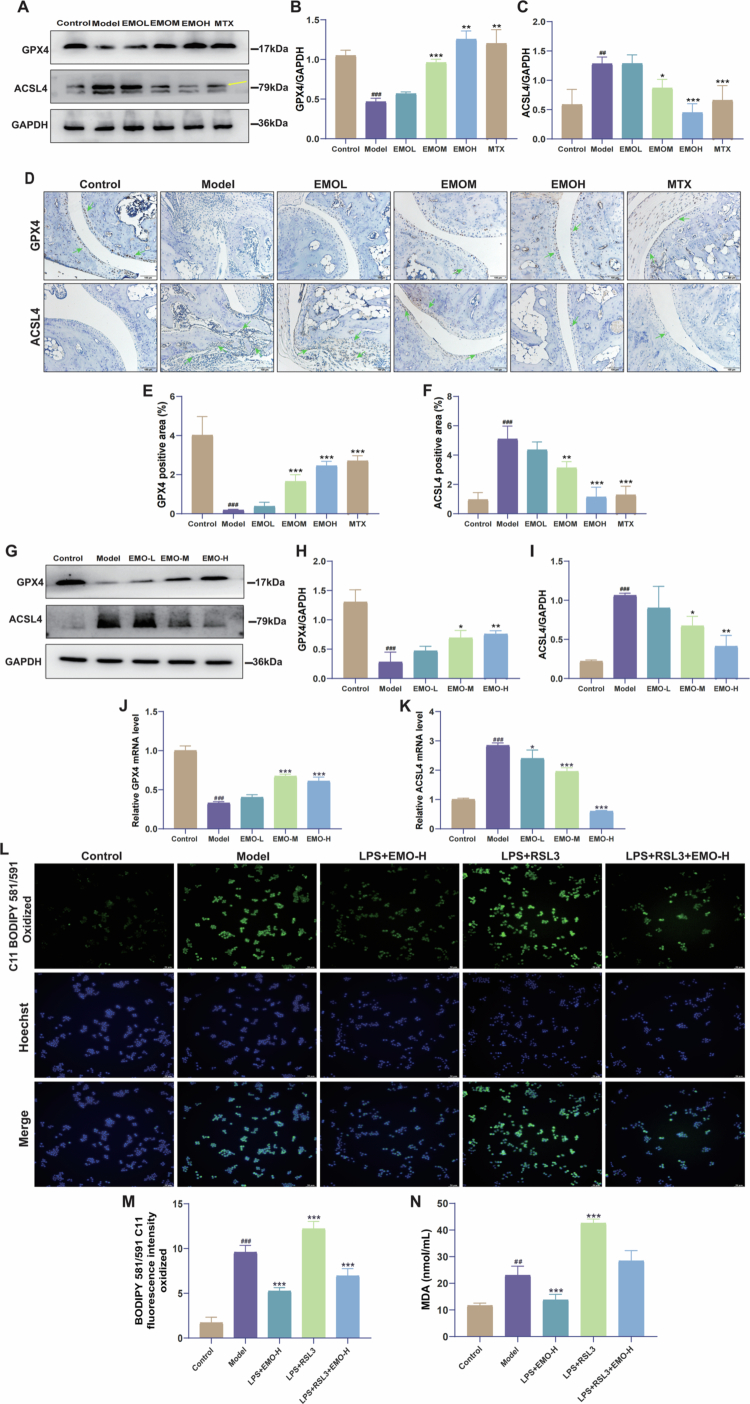
EMO inhibits macrophage ferroptosis via the GPX4/ACSL4 pathway to improve RA. (A–C) Representative western blot images and quantitative analysis of GPX4 and ACSL4 in CIA mice (n = 3); (D–F) Representative IHC images and quantitative analysis of GPX4 and ACSL4 (200×, n = 6); green arrow: indicates the region of positive protein expression; (G–I) Representative western blot images and quantitative analysis of GPX4 and ACSL4 in cells (n = 3). (J, K) GPX4 and ACSL4 mRNA expression (qPCR, n = 3) (L, M) Representative fluorescent images and quantitative analysis of cells stained with C11-BODIPY (200×, n = 3). (N) MDA content in each group (n = 3). Data are presented as mean ± SD. ^##^*p *< 0.01, ^###^*p *< 0.001 compared with the Control group; *p < 0.05, **p < 0.01, ***p < 0.001 compared with the Model group. Control group: normal mouse group or RAW264.7 macrophage group; Model group: CIA mouse group or LPS-induced RAW264.7 macrophage group; EMO-L group: low-dose emodin group; EMO-M group: medium-dose emodin group; EMO-H group: high-dose emodin group; MTX: methotrexate group; GPX4: glutathione peroxidase 4; ACSL4: acyl-CoA synthetase long-chain family member 4; IHC: immunohistochemistry.

Based on previous network pharmacology, proteomics, molecular docking, and MD simulation analyzes, we further validated our results in vivo and in vitro. To determine whether EMO inhibits ferroptosis via the GPX4/ACSL4 pathway, western blotting (Figure 9A–C) and IHC (Figure 9D–F) were performed. In contrast to the control group, the paw tissues of mice in the model group exhibited a significant decrease in GPX4 protein levels along with a notable increase in ACSL4 expression. EMO treatment effectively reversed these changes by upregulating GPX4 and downregulating ACSL4 expression.

In the LPS-induced RAW264.7 macrophage model, both qPCR and western blot analyszes showed that LPS stimulation downregulated GPX4 while upregulating ACSL4, consistent with activation of ferroptosis under inflammatory conditions. Following EMO treatment, GPX4 was significantly increased and ACSL4 was reduced at both the mRNA and protein levels, suggesting that EMO inhibited LPS-induced ferroptosis (Figure 9G–K).

To further confirm whether EMO suppresses ferroptosis via the GPX4/ACSL4 axis, the GPX4-specific inhibitor RSL3 was used to exacerbate ferroptosis in LPS-stimulated RAW264.7 macrophages. C11-BODIPY fluorescence analysis (Figure 9L, M) revealed that lipid peroxidation was moderately increased in the model (LPS) group compared with that in the control group and that this increase was significantly suppressed by EMO-H treatment. In contrast, the LPS + RSL3 group exhibited markedly enhanced lipid peroxidation, indicating aggravated ferroptosis. Notably, co-treatment with EMO-H in the LPS + RSL3 + EMO-H group substantially attenuated this RSL3-driven lipid peroxidation, restoring levels close to those observed in the LPS + EMO-H group. Consistently, MDA content followed a similar pattern (Figure 9N), with the highest levels observed in the LPS + RSL3 group and a significant reduction upon EMO-H co-administration. Collectively, these results demonstrate that EMO can counteract RSL3-aggravated ferroptosis, further supporting the conclusion that EMO inhibits ferroptosis by modulating the GPX4/ACSL4 axis, thereby alleviating macrophage damage associated with RA.

## 4. Discussion

The initiation and advancement of RA result from intricate interactions involving immune system dysfunction, chronic inflammation, metabolic irregularities, and ongoing cellular damage [25]. Although therapies targeting inflammatory mediators or immune signaling pathways have improved clinical outcomes, their ability to achieve sustained control of synovial injury and joint destruction remains limited, indicating that critical mechanisms underlying RA are not yet fully understood [26,27]. Therefore, identifying key cellular events that amplify tissue injury within the inflammatory microenvironment is essential, as this may help clarify RA pathogenesis and support the development of more effective preventive and therapeutic strategies.

Our previous work has shown that EMO exerts anti-inflammatory, antioxidant, and immunomodulatory effects [28]; however, its molecular mechanisms in RA pathogenesis remain poorly defined. In this research, we assessed the impact of EMO on pathology associated with RA by employing a CIA mouse model and LPS-activated RAW264.7 macrophages. EMO reduced joint inflammation and oxidative stress in CIA mice and improved histopathological changes in affected joints. In vitro, EMO dose-dependently attenuated LPS-induced injury in RAW264.7 cells, increased cell viability, and alleviated mitochondrial dysfunction. Mechanistically, EMO appeared to modulate the GPX4/ACSL4 axis and suppress macrophage ferroptosis, thereby contributing to the attenuation of RA-related tissue injury. These observations indicate that the protective effects of EMO extend beyond inflammation control and may involve regulation of programmed cell death under persistent inflammatory stress. Notably, by focusing on ferroptosis – a process increasingly implicated in RA but not yet fully characterized – this study provides additional mechanistic insight into how EMO may mitigate RA pathology. Collectively, this results endorse EMO as a promising therapeutic option for RA and offer a justification for additional exploration in more clinically pertinent models.

The progression of RA is marked by ongoing inflammation and an accumulation of oxidative stress. Persistent production of pro-inflammatory mediators, excessive ROS generation, and disrupted metabolic homeostasis within the synovial microenvironment collectively contribute to synovial cell injury [29-31]. Evidence indicates that ferroptosis, a regulated cell death pathway highly sensitive to redox imbalance and iron dysregulation, may contribute to the amplification of synovial injury under chronic inflammatory conditions [32,33]. The central event in ferroptosis is iron-driven lipid peroxidation, in which free Fe^2+^ promotes ROS generation via the Fenton reaction and accelerates peroxidation of polyunsaturated phospholipids, leading to the accumulation of membrane lipid hydroperoxides and loss of membrane integrity [34,35]. Disrupted iron metabolism is a key prerequisite for ferroptosis and is characterized by increased TFR1-mediated iron uptake and reduced FPN1-dependent iron export, expanding the labile iron pool and exacerbating oxidative injury [36]. In RA synovial tissue, pro-inflammatory cytokines such as TNF-α and IL-1β can suppress GSH synthesis, thereby limiting GPX4 activity and reducing clearance of lipid peroxides, which promotes lipid peroxidation and ferroptosis susceptibility [37–39]. Accordingly, increased ROS and MDA levels reflect oxidative stress and lipid peroxidation and may serve as surrogate indicators of ferroptosis vulnerability in the synovial microenvironment [40,41].

EMO treatment reduced MDA, labile Fe^2+^, and ROS levels while increasing GSH and SOD expression in both CIA mice and LPS-stimulated RAW264.7 macrophages, indicating that EMO enhances antioxidant capacity and attenuates lipid peroxidation, thereby mitigating inflammation-associated oxidative injury. In addition, EMO was associated with consistent regulation of key proteins involved in iron homeostasis. FTH1, a major intracellular iron-storage protein, can sequester labile iron and limit Fenton chemistry-driven ROS generation when upregulated [42,43]. Conversely, reduced TFR1 expression limits cellular iron uptake, thereby decreasing the labile iron pool and lowering susceptibility to ferroptosis [44,45]. HMOX1 plays context-dependent roles in ferroptosis: while moderate activity may support antioxidant responses, sustained or excessive activation can increase heme turnover and Fe^2+^ release, promoting lipid peroxidation and ferroptotic injury [46–48]. In both in vivo and in vitro models, an increase in FTH1 expression was observed with EMO treatment, while the abnormal upregulation of TFR1 and HMOX1 was inhibited. Overall, these results indicate that EMO alleviates ferroptosis by reestablishing iron homeostasis and reducing lipid peroxidation driven by labile iron.

RA progression is not governed by a single mode of cell death; rather, it involves coordinated contributions from multiple regulated cell death programs, including apoptosis, necroptosis, pyroptosis, and ferroptosis. Compared with apoptosis and pyroptosis, which are typically initiated by defined inflammatory cues, ferroptosis is particularly sensitive to cellular metabolic state, iron handling, and redox homeostasis. Consequently, ferroptosis may be more readily initiated and sustained in the chronically oxidized synovial microenvironment of RA, thereby exacerbating lipid peroxidation–mediated injury. Beyond inducing cellular dysfunction, ferroptosis may further sustain inflammation through lipid peroxidation and the release of DAMPs, creating a feed-forward loop linking oxidative stress, lipid peroxidation, and persistent inflammation. Previous studies suggest that EMO may also modulate other inflammation-associated cell death pathways; for example, EMO has been reported to inhibit HIF-1α/NLRP3 signaling and alleviate synovitis in RA models [49]. Therefore, suppression of ferroptosis likely represents one important component of the multi-target actions of EMO, rather than its sole mechanism of protection in RA.

In order to clarify the mechanisms through which EMO provides protective effects in RA, we integrated network pharmacology predictions with proteomic profiling to identify candidate pathways. Ferroptosis-related pathways were prominently enriched following EMO treatment, suggesting that ferroptosis may serve as a mechanistic entry point through which EMO modulates the inflammatory microenvironment in RA. GPX4 and ACSL4 emerged as key nodes potentially regulated by EMO within the ferroptosis network. To examine the interaction between EMO and these targets, molecular docking, MD simulations, and SPR assays were performed, supporting stable binding and favorable interaction profiles. In both CIA synovial tissue and LPS-stimulated RAW264.7 macrophages, EMO increased GPX4 expression while reducing ACSL4 levels, consistent with modulation of the GPX4/ACSL4 regulatory axis. To functionally validate this mechanism, RAW264.7 macrophages were exposed to the GPX4 inhibitor RSL3 to induce ferroptosis-like changes. EMO attenuated RSL3-induced lipid peroxidation and reduced MDA accumulation, indicating that EMO can partially counteract lipid oxidative damage when GPX4 activity is suppressed. Collectively, these findings demonstrate that EMO alleviates lipid peroxidation and ferroptosis by modulating the GPX4/ACSL4 axis, which may contribute to its anti-inflammatory and tissue-protective effects in RA.

Mitochondrial homeostasis may represent an additional mechanism underlying the protective effects of EMO. Mitochondria serve as central hubs for cellular metabolism and immune regulation and actively shape inflammatory signaling responses [50,51]. Increasing evidence indicates that mitochondrial dysfunction contributes to RA pathogenesis by affecting the survival, activation, and differentiation of immune and stromal cells, which in turn sustains chronic inflammation and promotes tissue injury [52]. Previous studies have shown that EMO protects against myocardial mitochondrial injury through activation of the PGC-1α pathway and attenuates ischemia/reperfusion-associated mitochondrial dysfunction [53,54], supporting a potential role for EMO in mitochondrial protection. In the CIA model, EMO improved mitochondrial ultrastructural alterations in inflamed joint tissues. In LPS-stimulated RAW264.7 macrophages, EMO restored MMP, reduced mtROS accumulation, and enhanced ATP production. These observations suggest that preservation of mitochondrial homeostasis may contribute to EMO's ability to limit oxidative stress and tissue injury in RA models. Considering the function of mitochondria in iron handling and lipid peroxidation, improving mitochondrial function may act synergistically with ferroptosis suppression to enhance the anti-inflammatory and tissue-protective effects of EMO.

Although this study was conducted in CIA mice and LPS-stimulated macrophages, accumulating evidence suggests that ferroptosis-related alterations may also be involved in human RA. Previous studies have reported increased lipid peroxidation, impaired antioxidant capacity, and dysregulated iron metabolism in synovial tissues from patients with RA, potentially accompanied by reduced GPX4 activity and heightened susceptibility to ferroptosis [55,56]. In addition, EMO can exert anti-inflammatory and antioxidant effects in human RA fibroblast-like synoviocytes and CD14⁺ macrophages [57]. Together, these observations provide indirect support for translational relevance; however, confirmation in patient-derived samples and primary human cells remains necessary. In the current study, the mechanistic interpretation mainly focused on ferroptosis effectors and downstream phenotypes, whereas upstream transcriptional regulation was not directly examined. Prior studies in other disease contexts indicate that EMO can activate Nrf2-dependent antioxidant responses [58] and may influence lipid metabolism through pathways such as PPARγ. Mechanistically, Nrf2 can regulate antioxidant defenses and may support GPX4 expression, whereas PPARγ and SREBP-dependent programs influence lipid remodeling and ferroptosis sensitivity [59]. Therefore, whether EMO modulates ferroptosis through these transcriptional networks warrants further investigation.

This study has some limitations. First, although the CIA model and LPS-stimulated macrophages capture key inflammatory features of RA, they do not fully recapitulate the immune heterogeneity of human disease. Second, our analyzes focused primarily on macrophage ferroptosis, and the effects of EMO on other pathogenic cell populations within the RA synovium, including fibroblast-like synoviocytes and T/B lymphocytes, remain to be elucidated. Additionally, the present study does not definitively establish whether ferroptosis is an early driver or a late amplifier of RA pathology; this temporal relationship requires further investigation. Finally, the pharmacokinetics, bioavailability, and safety profile of EMO were not characterized, limiting quantitative interpretation of exposure–response relationship and translational evaluation. Future studies should incorporate patient-derived samples, multi-cell validation platforms, and systematic pharmacological evaluations to strengthen the translational relevance of EMO in RA.

## 5. Conclusions

This research illustrates that EMO exerts anti-RA effects by inhibiting ferroptosis. Our findings both extend our comprehension of the link between RA and ferroptosis, and position EMO as a promising ferroptosis-targeting therapeutic candidate, offering a novel theoretical foundation for the development of precision treatments for RA.

## Supplementary Material

Supplementary material Supplementary Material

Supplementary material ARRIVE checklist.pdf

## Data Availability

All the data generated or analyzed during this study are included in this published article (and its Supplementary Data files). The proteomics raw datasets have also been deposited in the iProX (PXD number: PXD074847).
