# Supplementary material for: Ferroptosis inhibition and mitochondrial rescue: a novel mechanism of emodin in rheumatoid arthritis
Source: Redox Rep. 2026 Mar 22;31(1):2646383. doi: 10.1080/13510002.2026.2646383 (PMC13007403; doi:10.1080/13510002.2026.2646383)
Supplement: Supplementary material [file YRER_A_2646383_SM2359.docx]

**SUPPLEMENTARY DATA**

**Ferroptosis inhibition and mitochondrial rescue: A novel mechanism of emodin in rheumatoid arthritis**

Linlan Zhou^1^^†^, Jun Liu^2†^, Jing Ren^3†^, Dehao Du^1^, Xiaofeng Rong^1^*

^1^ Department of Combination of Chinese and Western Medicine, The First Affiliated Hospital of Chongqing Medical University, Chongqing 400016, China

^2^ Department of Rehabilitation Medicine of Jiangbei Campus, The First Affiliated Hospital of Army Medical University, Chongqing, 400020, China

^3^ College of Traditional Chinese Medicine, Chongqing Medical and Pharmaceutical College, Chongqing 401331, China

***Corresponding author**: Xiaofeng Rong, The First Affiliated Hospital of Chongqing Medical University, Chongqing 400016, China; E-mail: [cyrxf202194@163.com](mailto:cyrxf202194@163.com)

E-mail addresses: 545291299@qq.com (L.L. Zhou), [17843995590@163.com](mailto:17843995590@163.com) (J. Liu), [15320450137@163.com](mailto:15320450137@163.com) (J. Ren), [1811988385@qq.com](mailto:1811988385@qq.com) (D.H. Du), [cyrxf202194@163.com](mailto:cyrxf202194@163.com) (X.F. Rong)

^†^ These authors contributed equally to this work.

**Supplementary Data**

**2. Materials and Methods**

**2.8 Western blot**

**2.8.1 Protein extraction and quantification**

Proteins were extracted from murine tissues (synovium and periarticular soft tissues of the hind paws) and cultured cells using RIPA lysis buffer supplemented with 1% PMSF (ST506, Beyotime, China). Concentrations were quantified using a BCA assay kit (P0010S, Beyotime, China ).

**2.8.2 Western blotting**

Equal protein amounts were separated by SDS-PAGE and electrophoretically transferred to PVDF membranes (IPVH00010, Merck, Germany). Membranes were blocked with 5% non-fat milk in TBST for 1 hour at room temperature. Incubation with primary antibodies was performed overnight at 4°C. After washing with TBST, membranes were incubated with an HRP-conjugated secondary antibody for 1 hour at room temperature.

**2.8.3 Detection and analysis**

Blots were developed using a Bio-Rad ChemiDoc imaging system (12003153,Bio-Rad Laboratories, USA) under chemiluminescence mode. Densitometric analysis of the bands was conducted using ImageJ software (NIH).

**Note: Primary antibodies, working dilutions, incubation conditions, and secondary antibodies are detailed in Supplementary Table 1.**

*** Supplementary Table 1**

| **Primary antibody** | **Application** | **Host/Clonality** | **Supplier** | **Catalog #** | **Working dilution** | **Incubation** | **Secondary (brand/cat#)** |
| --- | --- | --- | --- | --- | --- | --- | --- |
| TFR1 | Western blot (WB) | Rabbit monoclonal | Abmart (Shanghai, China) | T56618 | 1:1000 | Overnight at 4℃ | HRP anti-rabbit IgG (H+L)，SA00001-2，Proteintech (Wuhan, China) |
| FTH1 | WB | Rabbit | Abmart (Shanghai, China) | T55648 | 1:1000 | Overnight at 4℃ | HRP anti-rabbit IgG (H+L)，SA00001-2，Proteintech (Wuhan, China) |
| HMOX1 | WB | Rabbit polyclonal | Proteintech (Wuhan, China) | 10701-1-AP | 1:10000 | Overnight at 4℃ | HRP anti-rabbit IgG (H+L)，SA00001-2，Proteintech (Wuhan, China) |
| GAPDH | WB (loading control) | Rabbit polyclonal | Proteintech (Wuhan, China) | 10494-1-AP | 1:20000 | overnight at 4℃ | HRP anti-rabbit IgG (H+L)，SA00001-2，Proteintech (Wuhan, China) |
| ACSL4 | WB | Rabbit | Abcam (Cambridge, UK) | AB155282 | 1:10000 | Overnight at 4℃ | HRP anti-rabbit IgG (H+L)，SA00001-2，Proteintech (Wuhan, China) |
| GPX4 | WB | Rabbit | Abcam (Cambridge, UK) | AB125066 | 1:10000 | Overnight at 4℃ | HRP anti-rabbit IgG (H+L)，SA00001-2，Proteintech (Wuhan, China) |

**2.8.4 Original Western blot images**

**
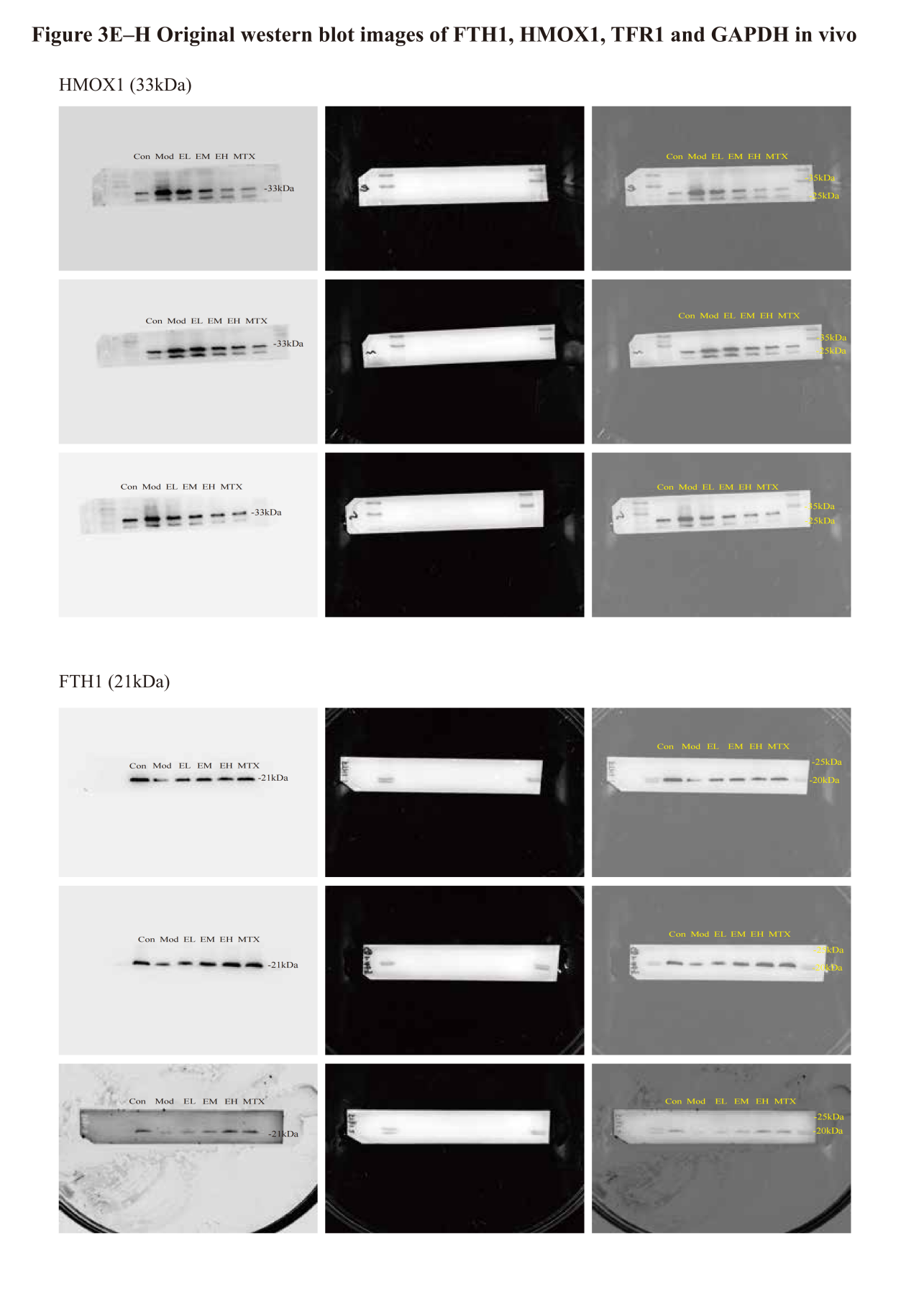
**

**
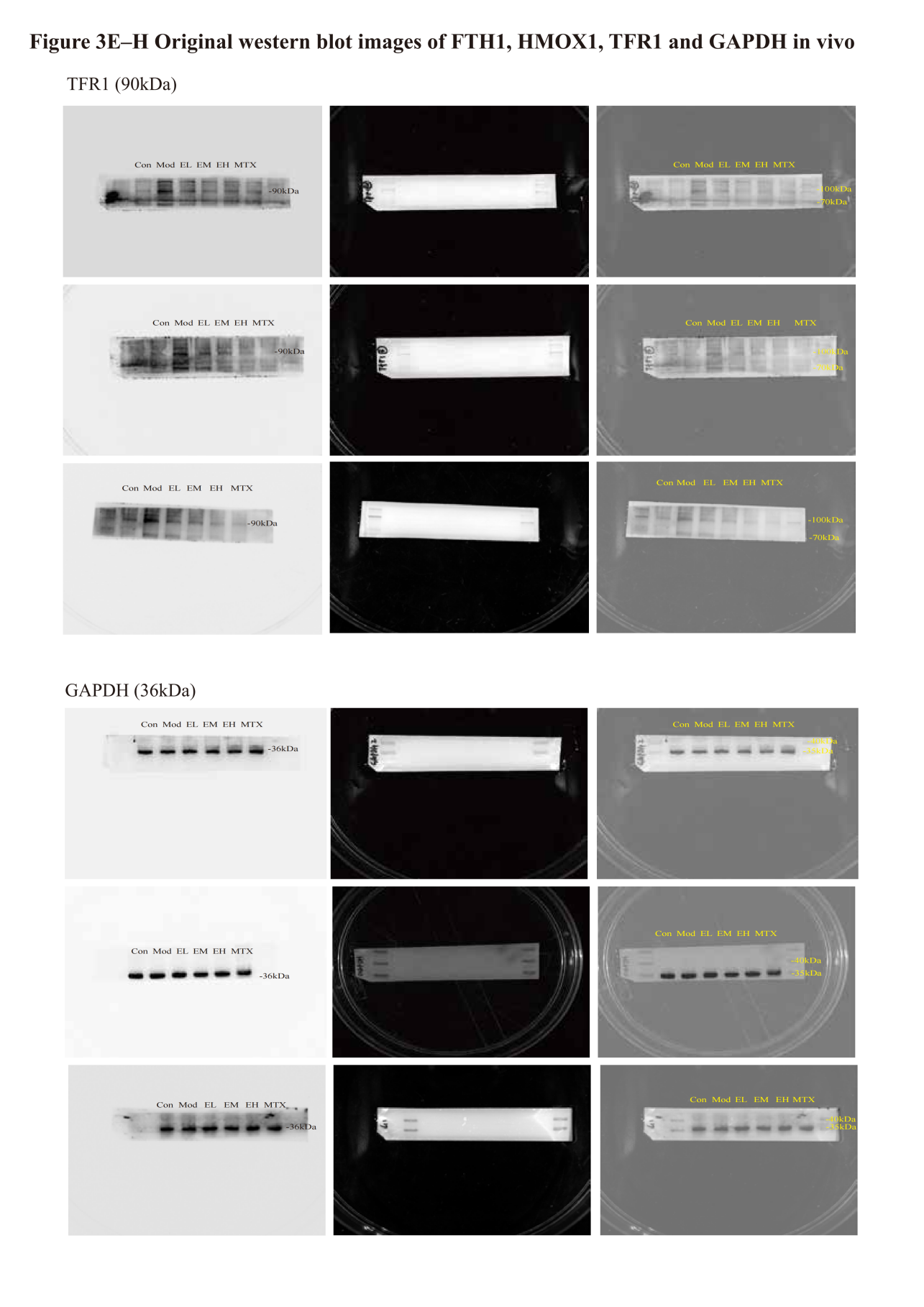
**

**
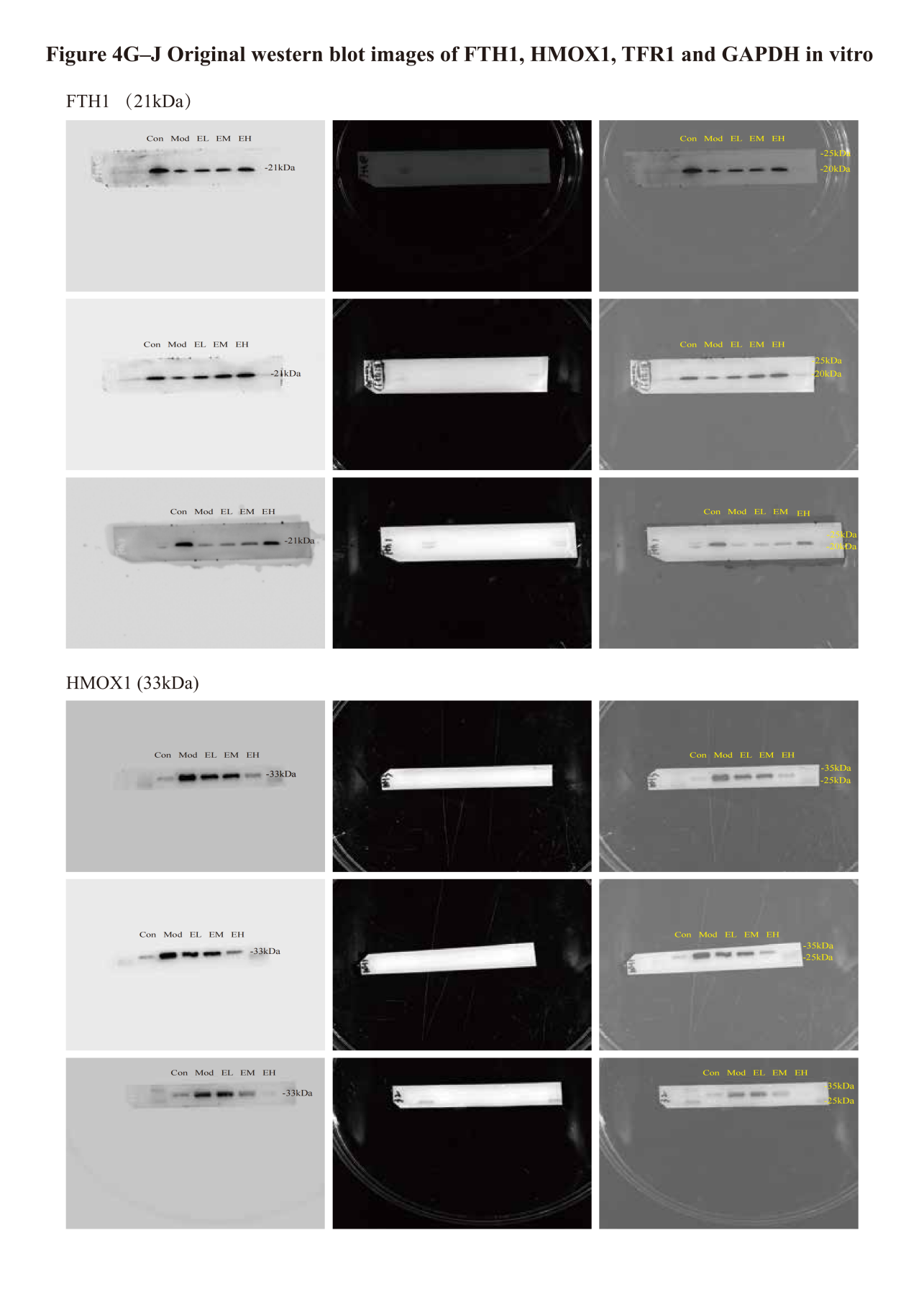
**

**
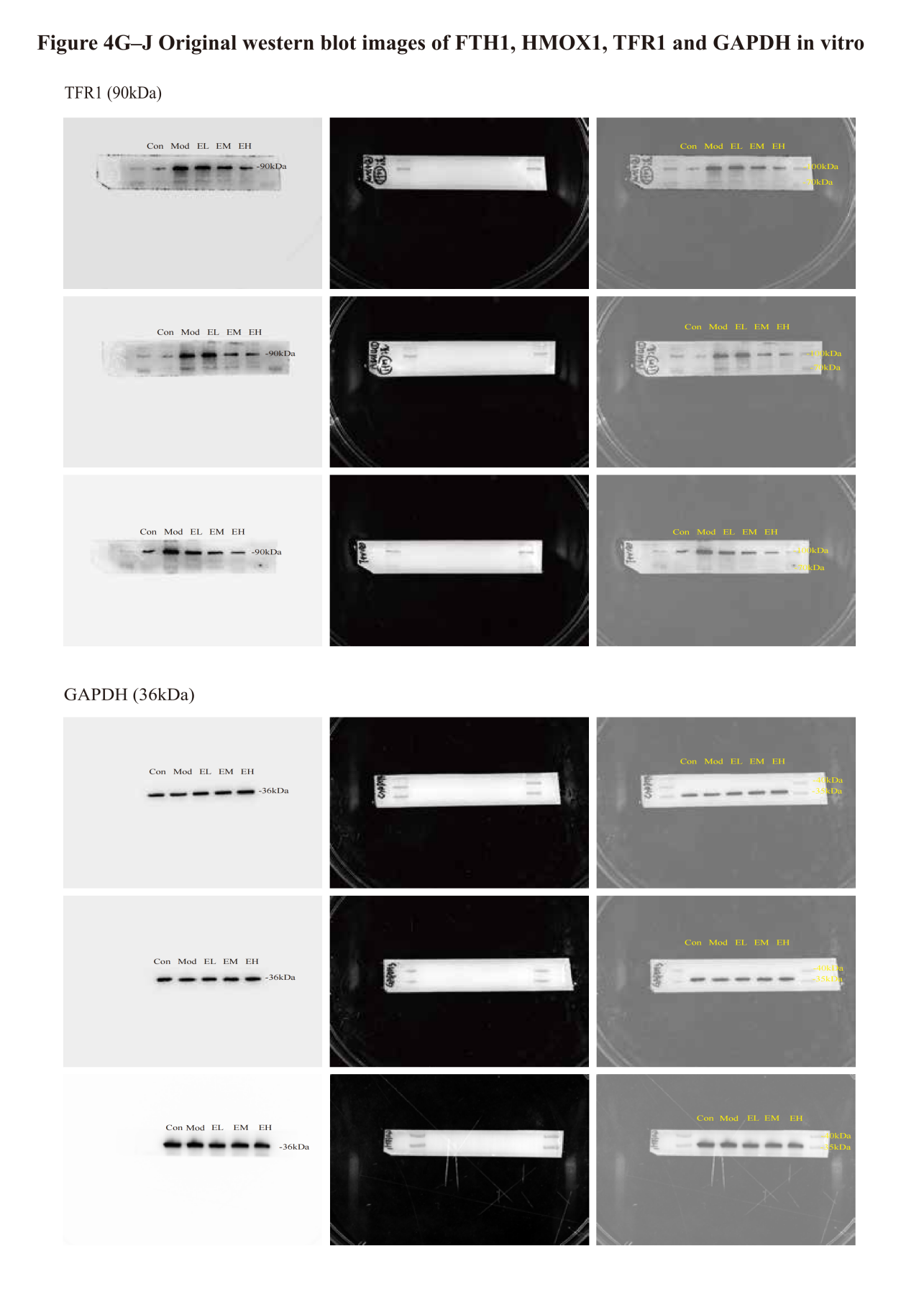
**

**
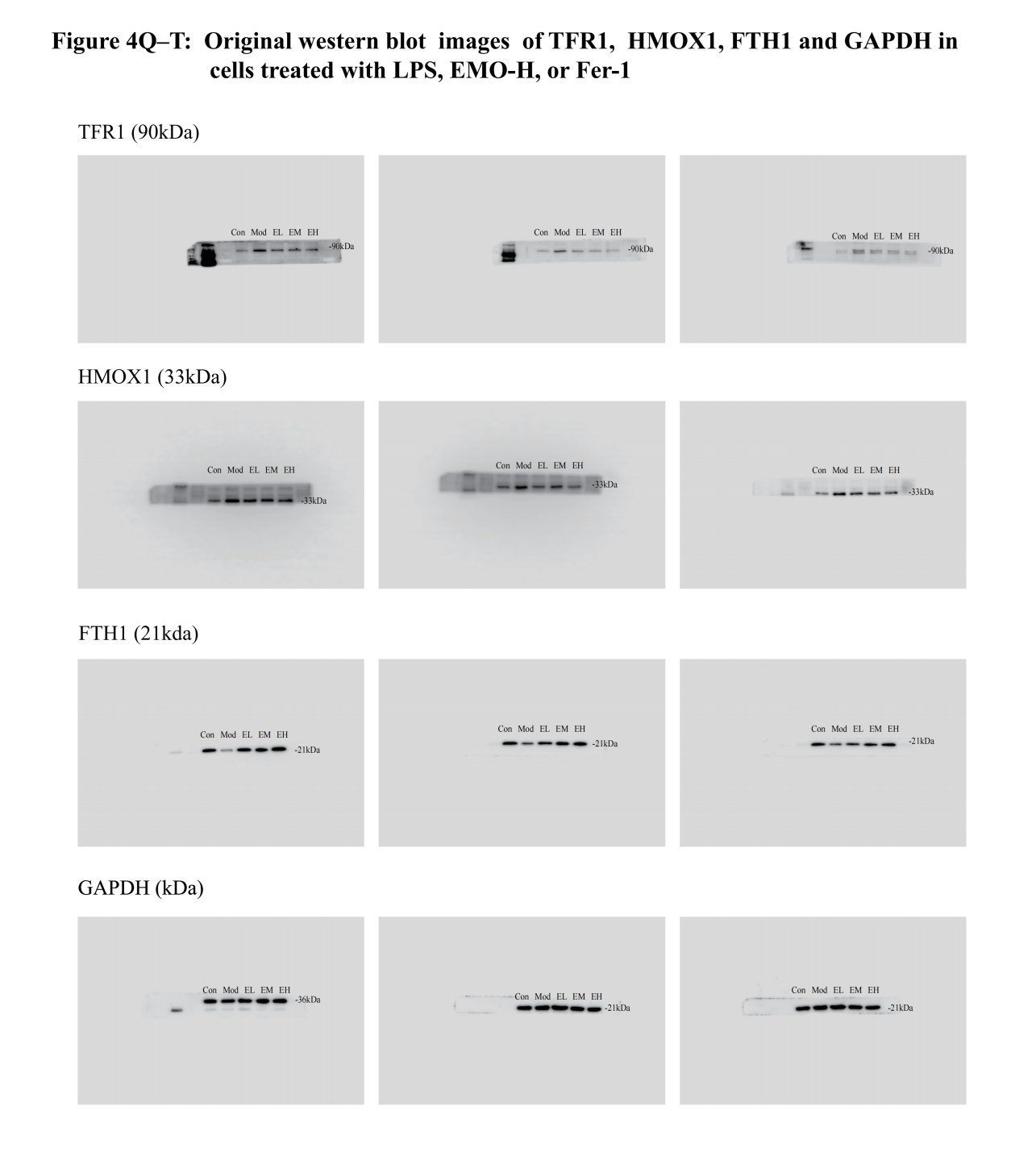
**

**
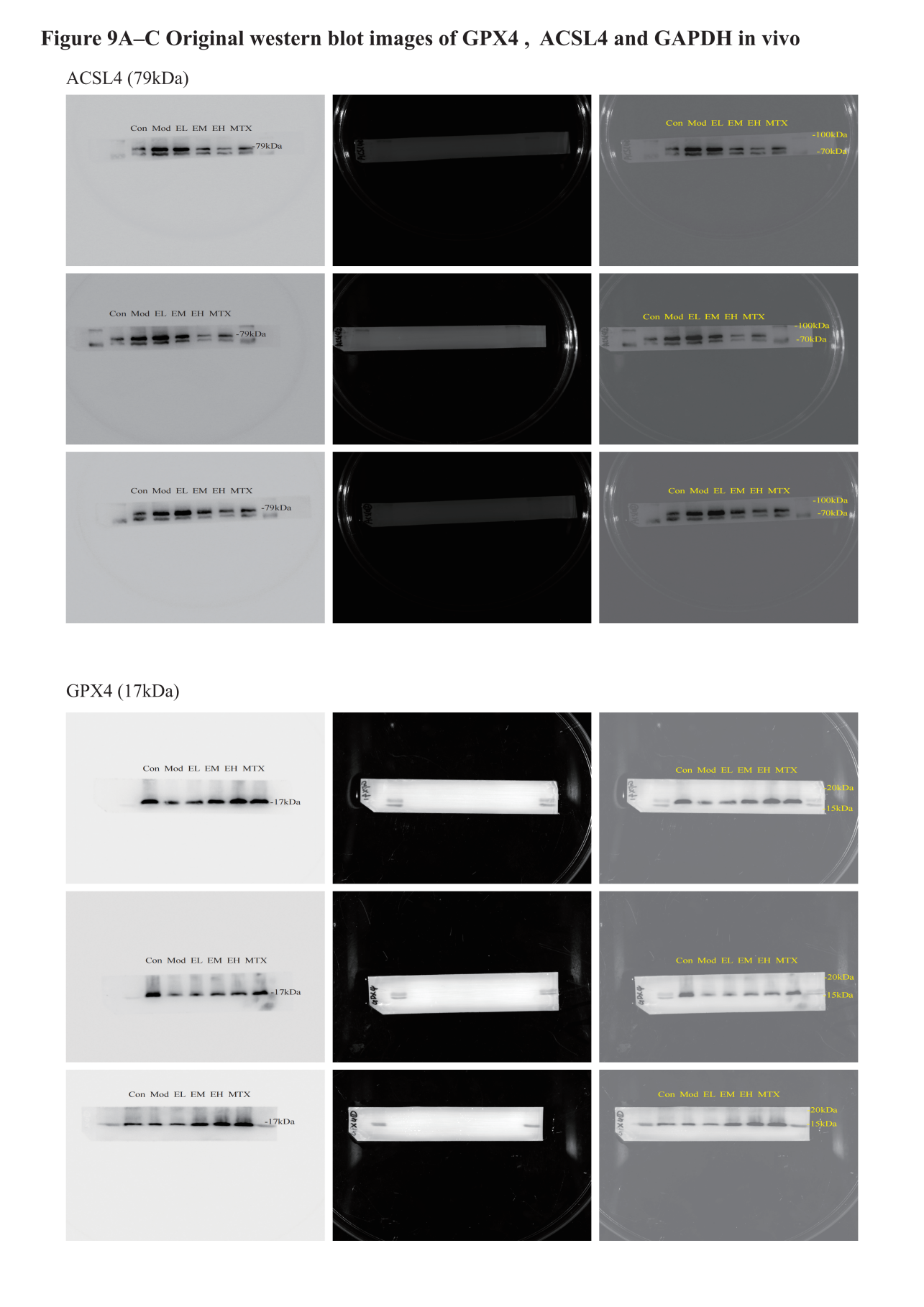
**

**
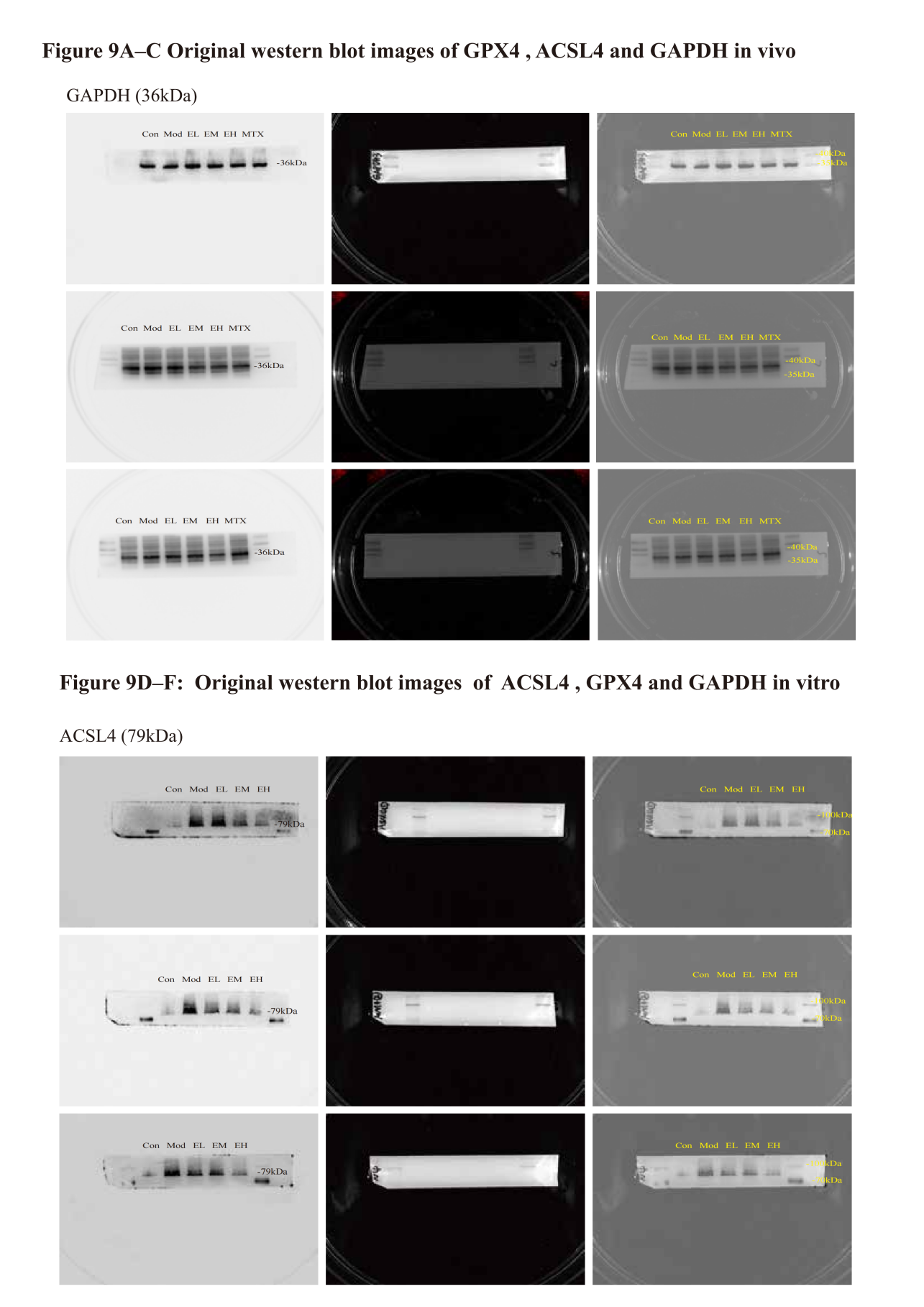
**

**
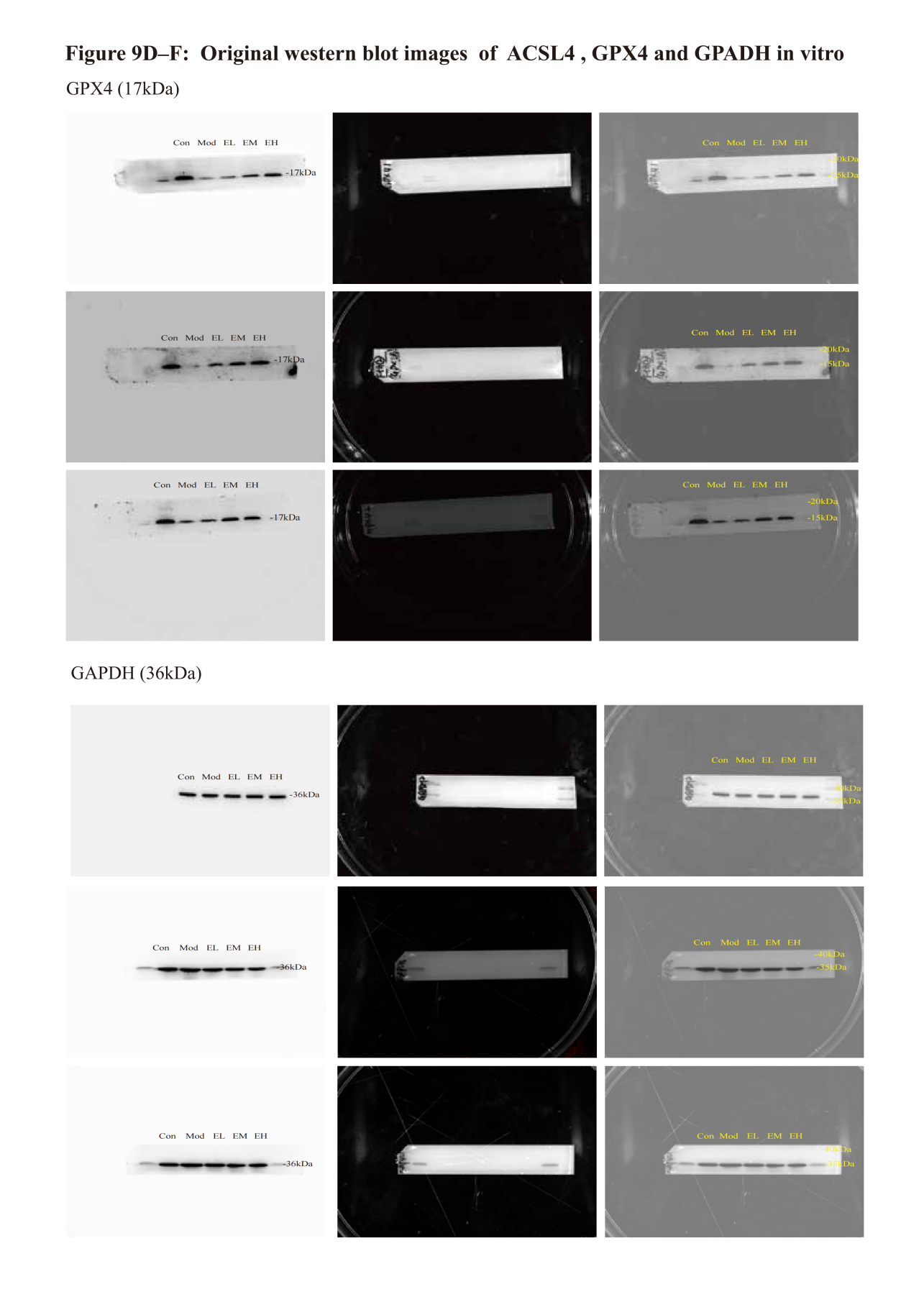
**

**2.9 Immunohistochemistry (IHC) analysis**

**2.9.1 Tissue preparation**

Hind paw joints from mice were fixed in 4% paraformaldehyde, dehydrated through a graded ethanol series, cleared in xylene, and embedded in paraffin blocks. Subsequently, 4-5 μm thick sections were cut using a microtome and mounted onto glass slides for immunohistochemical processing.

**2.9.2 Immunohistochemical staining**

Following deparaffinization, rehydration, and antigen retrieval, sections were blocked with a solution containing normal serum and 1% BSA to minimize non-specific binding. The sections were then incubated overnight at 4°C with a panel of primary antibodies targeting proteins central to ferroptosis and iron homeostasis, namely:Anti-GPX4 (AF301385, 1:100, AiFang, China), Anti-ACSL4 (AB155282, 1:100, Abcam, MA, USA), Anti-Ferritin Heavy Chain (FTH1) (4393, 1:200, Cell Signal, China), Anti-TFR1 (AF16541, 1:100, AiFang, China), Anti-Heme Oxygenase 1 (HMOX1) (AF11845, 1:50, AiFang, China)

**2.9.3 Signal detection and visualization**

After washing to remove unbound primary antibodies, the immunoreactivity was visualized by incubating the sections with a species-appropriate biotinylated secondary antibody, followed by an avidin-biotin-peroxidase complex (ABC). The peroxidase activity was developed using a 3,3'-Diaminobenzidine (DAB) chromogen substrate, which yields an insoluble brown deposit at the sites of antibody binding. The sections were then counterstained with hematoxylin to provide nuclear contrast.

**2.9.4 Image acquisition and quantitative analysis**

Whole-slide images were captured at 200× magnification under bright-field illumination using an Olympus BX53 microscope equipped with a high-resolution digital camera. The digital images were processed and quantified using ImageJ software (National Institutes of Health) to measure the percentage of positive cells.

**Note: Primary antibodies, working dilutions, incubation conditions, and secondary antibodies are detailed in Supplementary Table 2.**

*** Supplementary Table 2**

| **Target** | **Application** | **Host/Clonality** | **Supplier** | **Catalog #** | **Working dilution** | **Incubation** | **Secondary (brand/cat#)** |
| --- | --- | --- | --- | --- | --- | --- | --- |
| GPX4 | Immunohistochemistry (IHC) | Rabbit | AiFang (China) | AF301385 | 1:100 | Overnight at 4℃ | Biotinylated/HRP per kit，SA00001-2，Proteintech (Wuhan, China) |
| ACSL4 | IHC | Rabbit | Abcam (MA, USA) | AB155282 | 1:100 | Overnight at 4℃ | Biotinylated/HRP per kit，SA00001-2，Proteintech (Wuhan, China) |
| FTH1 | IHC | Rabbit | Cell Signaling | #4393 | 1:200 | Overnight at 4℃ | Biotinylated/HRP per kit，SA00001-2，Proteintech (Wuhan, China) |
| TFR1 | IHC | Rabbit | AiFang (China) | AF16541 | 1:100 | Overnight at 4℃ | Biotinylated/HRP per kit，SA00001-2，Proteintech (Wuhan, China) |
| HMOX1 | IHC | Rabbit | AiFang (China) | AF11845 | 1:50 | Overnight at 4℃ | Biotinylated/HRP per kit，SA00001-2，Proteintech (Wuhan, China) |

**2.13. Quantitative real-time PCR (qPCR)**

Total RNA was extracted using an RNA Extraction Kit (TSP413, Qinke Biotechnology, China). Subsequently, 1 µg of total RNA was reverse-transcribed into cDNA using the SynScript® Ⅲ RT SuperMix for qPCR kit (DLR101, Daling Biotechnology, China). Quantitative real-time PCR (qPCR) was performed on an ABI QuantStudio™ Plus system. Each 20 µL reaction mixture contained 10 µL SYBR Green mix, 0.8 µL each of forward and reverse primers (10 µM), 1 µL diluted cDNA, and 7.4 µL ddH2O. The thermocycling conditions consisted of an initial denaturation at 95 °C for 5 min, followed by 40 cycles of denaturation at 95 °C for 15 s, and annealing/extension at 60 °C for 15 s, with fluorescence signal acquisition. A final melting curve analysis was performed by gradually increasing the temperature to 95 °C. All samples were run in triplicate. Relative gene expression levels were calculated using the 2^−ΔΔCt^ method, with GAPDH as the internal control.

**Note: Primer sequences, accession code and amplicon size are detailed in Supplementary Table 3.**

*** Supplementary Table 3**

| **Gene Name** | **Accession Code** | **Primer Sequence (5‘ → 3’)** | **Amplicon Size (bp)** |
| --- | --- | --- | --- |
| Gpx4 | NM-008162.4 | F: TTGATAAGAACGGCTGCGTG  R: GGCACACACTTGTAGGGCT | 107 |
| Acsl4 | NM-019477.3 | F: TTCGAGAAGCTGCAAATGCC  R: TGTTACCAAACCAGTCTCGGG | 101 |
| Fth1 | NM-010239.2 | F: CTGGAACTGCACAAACTGGC  R: CTTCAGGGGCACCCATCTTG | 145 |
| Hmox1 | NM-010442.2 | F: AGACCAGAGTCCCTCACAGA  R: GAGCTAGTGCTGATCTGGGG | 111 |
| Tfr1 | NM-011638.4 | F: CCGCTCGTGGAGACTACTTC  R: GGAGATACATAGGGCGACAGG | 145 |
| Gapdh | NM-008084.4 | F: ACTCTTCCACCTTCGATGCC  R: TGGGATAGGGCCTCTCTTGC | 193 |

**2.18 Proteomics analysis**

Following EMO treatment, RAW264.7 cells were lysed in RIPA buffer, and cell debris was removed by ultracentrifugation to obtain total protein. Protein concentrations were quantified using the BCA method, and protein samples were separated by SDS–PAGE. Proteins were digested with trypsin and subsequently purified using solid-phase extraction.

Purified peptide samples were analyzed using a high-resolution liquid chromatography–tandem mass spectrometry system (LC–MS/MS; EASY-nLC 1200 coupled to an Orbitrap Exploris 480) operated in data-independent acquisition (DIA) mode. For protein identification, the acquired spectra were searched against the UniProt mouse reference proteome database (Mus_musculus_10090_SP_20230103.fasta) using the DIA-NN software (version 1.8). To control for false positives, a false discovery rate (FDR) threshold was set to < 1% at both the peptide and protein levels. Label-free quantification was performed based on extracted ion chromatograms of precursor peptides to determine changes in protein expression.

Statistical analysis was then conducted to identify differentially expressed proteins (DEPs). Proteins were considered significantly altered if they met the following dual criteria: a fold change (ratio) ≥ 1.5 (or ≤ 0.67) and a P-value < 0.05. The resulting DEPs were subjected to functional annotation, and pathway enrichment and protein–protein interaction (PPI) network analyses were performed using bioinformatics tools to elucidate key EMO-mediated molecular mechanisms.

**2.19 Network pharmacology**

The compound EMO was retrieved from the PubChem database, and its chemical structure files (SDF and SMILES formats) were downloaded. Potential protein targets of EMO were predicted using three complementary databases: PharmMapper (fit score ≥ 0.8), SwissTargetPrediction (probability ≥ 0.7), and SEA Search (Max TC ≥ 0.7). All target predictions were restricted to Homo sapiens to ensure biological relevance. RA-related targets were collected from the GeneCards (relevance score ≥ 10) and DisGeNET (score ≥ 0.3) databases using “rheumatoid arthritis” as the keyword. The intersection between EMO-predicted targets and RA-related targets was identified using Venny 2.0 software, yielding the final candidate target set after duplicate removal. This multi-step filtering strategy ensured the identification of high-confidence targets, including ACSL4 and GPX4, for subsequent analyses. A PPI network of the common targets was constructed using the STRING database and visualized with Cytoscape 3.9.1. Potential mechanisms underlying the therapeutic effects of EMO against RA were further explored through Gene Ontology (GO) and Kyoto Encyclopedia of Genes and Genomes (KEGG) pathway enrichment analyses.

**2.20. Molecular docking and molecular dynamics (MD) simulations**

**2.20.1 Molecular docking**

Molecular docking was performed to predict the binding affinity and interactions between the candidate compound and the key targets (ACSL4 and GPX4). The three-dimensional structures of ACSL4 and GPX4 were obtained from the RCSB PDB database. The ligand structure was retrieved from PubChem in SDF format and converted to PDB format using OpenBabel. Protein preparation, including water removal, hydrogen addition, and charge assignment, was conducted using AutoDock Tools 1.5.6. Both the ligand and receptor were then converted into the PDBQT format. Molecular docking was carried out using AutoDock. The docking results were visualized and analyzed using PyMOL and LigPlot software.

**2.20.2 Molecular dynamics (MD) simulations**

Molecular dynamics simulations were performed to assess the stability and dynamic interactions of the protein-ligand complexes using GROMACS 2020.3. The initial structures were the top-ranked docking poses. The protein was described by the amber99sb-ildn force field, while ligand parameters were generated using the General Amber Force Field (GAFF). The system was solvated in a cubic box with SPC216 water molecules, maintaining a minimum distance of 1.0 nm between the protein and the box boundary. Na⁺ and Cl⁻ ions were added to neutralize the system charge. Energy minimization was conducted using the steepest descent algorithm. The system was then equilibrated under the NVT ensemble for 100 ps at 300 K, followed by the NPT ensemble for 100 ps at 1 bar. A production MD simulation of 50 ns was subsequently performed with a time step of 2 fs under periodic boundary conditions. The V-rescale thermostat and Parrinello-Rahman barostat were used to maintain temperature and pressure, respectively. Long-range electrostatic interactions were treated using the Particle Mesh Ewald (PME) method, and all bonds were constrained using the LINCS algorithm. Trajectory analysis, including calculations of root-mean-square deviation (RMSD), root-mean-square fluctuation (RMSF), solvent-accessible surface area (SASA), radius of gyration (Rg), and hydrogen bond occupancy, was performed using GROMACS utilities. The binding free energy was calculated using the gmx_mmpbsa tool based on frames extracted from the equilibrated phase of the simulation. Visualization and analysis of trajectories were conducted using VMD 1.9.3 and PyMOL 2.4.1.

**2.21 Surface plasmon resonance (SPR) binding assay**

SPR experiments were conducted using a Biacore system (Cytiva). The CM5 sensor chip (BR‑1005‑30, Cytiva) was installed according to the manufacturer's instructions. Before protein immobilization, the chip surface was activated by injecting a 1:1 mixture of 0.4 M EDC and 0.1 M NHS from the Aminocoupling Kit, (BR100050, Cytiva) over flow cells 2 and 4 for 7 min at a flow rate of 10 μL/min. Recombinant human GPX4 and ACSL4 proteins were separately diluted to 50 μg/mL in acetate buffer (pH 4.5; BR100349, Cytiva) and injected over the activated surfaces: GPX4 was immobilized on flow cell 2 and ACSL4 on flow cell 4, with immobilization levels reaching approximately 10,000 RU. After ligand coupling, residual active esters were blocked by injecting 1 M ethanolamine‑HCl (pH 8.5) for 7 min at 10 μL/min. Flow cells 1 and 3 were treated identically but without protein injection to serve as reference channels. Binding assays were performed at 25 °C using a running buffer consisting of 1× PBS‑P⁺ (pH 7.4; 28995084, Cytiva) containing 5% (v/v) DMSO (D8418, Sigma, USA). To correct for solvent‑induced refractive index changes, a solvent calibration curve was prepared by mixing 4.5% and 5.8% DMSO solutions according to a predefined gradient. EMO was serially diluted in the running buffer and injected over the chip surfaces at a flow rate of 30 μL/min for 60 s, followed by a 300‑s dissociation phase. After each injection cycle, the chip surface was regenerated with 10 mM glycine‑HCl (pH 2.0) for 5 min to remove bound analyte.

Sensorgrams were recorded in real time and processed using Biacore Insight Evaluation Software (Cytiva). Data were double‑referenced against both reference flow cells and blank buffer injections. Binding kinetics were globally fitted to a 1:1 Langmuir binding model to obtain the equilibrium dissociation constant (K_D_).
